# Systems based analysis of human embryos and gene networks involved in cell lineage allocation

**DOI:** 10.1186/s12864-019-5558-8

**Published:** 2019-03-05

**Authors:** H. L. Smith, A. Stevens, B. Minogue, S. Sneddon, L. Shaw, L. Wood, T. Adeniyi, H. Xiao, P. Lio, S. J. Kimber, D. R. Brison

**Affiliations:** 1 Maternal and Fetal Health Research Centre, University of Manchester, Manchester Academic Health Sciences Centre, Oxford Road, Manchester, M13 9WL UK; 2 Division of Developmental Biology & Medicine, School of Medical Sciences, University of Manchester, Manchester Academic Health Sciences Centre, 5th Floor Research, Royal Manchester Children’s Hospital, Oxford Road, Manchester, M13 9WL UK; 3 Division of Cell Matrix Biology and Regenerative Medicine, School of Biological Sciences, Faculty of Biology, Medicine and Health, University of Manchester, Manchester Academic Health Sciences Centre, Oxford Road, Manchester, M13 9WL UK; 4 Department of Reproductive Medicine, Saint Mary’s Hospital, Manchester University NHS Foundation Trust, Manchester Academic Health Sciences Centre, Oxford Road, Manchester, M13 9WL UK; 5 Computer Laboratory, William Gates Building, University of Cambridge, Cambridge, UK

**Keywords:** Human embryos, Gene networks, Cell lineage, Blastomere

## Abstract

**Background:**

Little is understood of the molecular mechanisms involved in the earliest cell fate decision in human development, leading to the establishment of the trophectoderm (TE) and inner cell mass (ICM) stem cell population. Notably, there is a lack of understanding of how transcriptional networks arise during reorganisation of the embryonic genome post-fertilisation.

**Results:**

We identified a hierarchical structure of preimplantation gene network modules around the time of embryonic genome activation (EGA). Using network models along with eukaryotic initiation factor (EIF) and epigenetic-associated gene expression we defined two sets of blastomeres that exhibited diverging tendencies towards ICM or TE. Analysis of the developmental networks demonstrated stage specific EIF expression and revealed that histone modifications may be an important epigenetic regulatory mechanism in preimplantation human embryos. Comparison to published RNAseq data confirmed that during EGA the individual 8-cell blastomeres are transcriptionally primed for the first lineage decision in development towards ICM or TE.

**Conclusions:**

Using multiple systems biology approaches to compare developmental stages in the early human embryo with single cell transcript data from blastomeres, we have shown that blastomeres considered to be totipotent are not transcriptionally equivalent. Furthermore we have linked the developmental interactome to individual blastomeres and to later cell lineage. This has clinical implications for understanding the impact of fertility treatments and developmental programming of long term health.

**Electronic supplementary material:**

The online version of this article (10.1186/s12864-019-5558-8) contains supplementary material, which is available to authorized users.

## Background

The preimplantation period is a unique window in development, when extensive remodelling of the genome and epigenome occur over the few days that the embryo is a free-living entity. This window is of critical importance allowing the fertilised oocyte to (i) correctly reprogram the male genome and activate the new embryonic genome, (ii) substantially reset the epigenome via a process that includes generalised demethylation and remethylation, and (iii) establish the first cell fate decision in development towards formation of the trophectoderm (TE), while maintaining the inner cell mass (ICM) stem cell population [1–3].

The processes underlying some of the key molecular and cellular decisions are not fully understood, including Embryonic Genome Activation (EGA), mechanisms underlying epigenome remodelling, and cell fate decisions governing blastomere development and differentiation into the ICM or trophectoderm (TE) tissues of the blastocyst. Early embryos inherit stored mRNA and proteins from the oocyte which guide development under maternal control until EGA. The degradation of particular inherited mRNAs may itself regulate the timing of EGA [4]. The start of EGA in the human was originally defined as occurring between the 4-cell and 8-cell stage [5]. However more recent studies have suggested that there are in fact three waves of EGA, occurring at the 2 cell, 4-cell and 8–10 cell stages, with the final representing the highest level of transcriptional activity [6]. Epigenetic remodelling in the human embryo occurs post fertilisation, with embryonic DNA generally demethylated to 30–40% by the 2-cell stage, with minimal subsequent loss of methylation, and no re-methylation until the post-implantation stages [7]. During this period of intensive remodelling, cell fate decisions are initiated which lead to segregation of human ICM and TE. It is currently unknown as to whether individual blastomeres from 8-cell embryos are pre-determined to become ICM or TE, but it is known that considerable heterogeneity exists between blastomeres at this point, due in part to the absence of gap junctional communication prior to this stage [8, 9]. The reported variation in different transcript subsets between individual human 8-cell blastomeres has been interpreted as indicating that some may arrest whilst others develop [10]. In particular, individual 8-cell blastomeres were reported to display significant variation in down-regulation of maternally-expressed genes, which may indicate early lineage specification. In contrast, other studies have shown that blastomeres from 5- to 8-cell human embryos are not pre-determined to become either ICM or TE [11]. We hypothesised that an analysis of the global human embryo transcriptome using a combination of network analyses, dimensional scaling and change point analysis would allow us to identify the transcriptional co-ordination involved in the key early developmental event of blastomere cell fate specification.

## Methods

### Oocytes and embryos

**Fig. 1:**
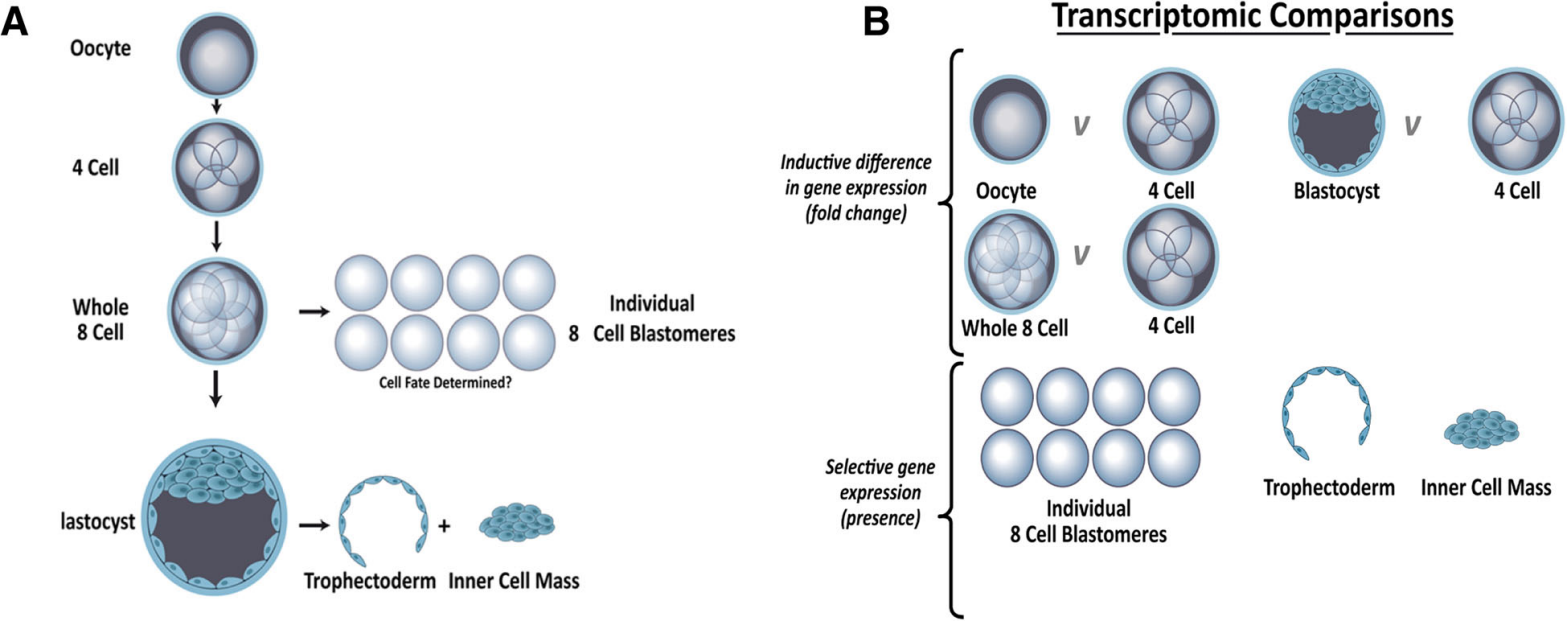
Schematic representing the human embryonic stages analysed and transcriptomic comparisons. **a**) The human embryonic stages analysed. We carried out single embryo and blastomere polyA-PCR global transcriptome profiling on replicate oocytes and embryos at the 4-cell (*n* = 4), 8-cell (*n* = 3) and blastocyst (*n* = 4) stage, together with 8 individual blastomeres disaggregated from one 8-cell embryo, and trophectoderm (*n* = 4) and inner cell mass (*n* = 4) disaggregated from blastocysts. We carried out single embryo and blastomere polyA PCR and qPCR on 24 individual blastomeres disaggregated from 3 complete sets. 8 individual blastomeres were used for qPCR validation. **b**) Different transcriptomic comparisons applied throughout the paper. To determine differences in gene expression between samples ANOVA was performed on 4-cell embryos vs. oocytes, 8-cell embryos and blastocysts. Benjamini-Hochberg false discovery rate (FDR) corrected *p*-values ≤0.05 were considered differentially expressed. Frozen robust multi-array analysis (fRMA) was used to define absolute expression by comparison to publically available microarray datasets within R and an expression barcode was defined for the 8-cell blastomere, ICM and TE sample

Human oocytes and embryos were donated to research with fully informed patient consent in writing and approval from South Manchester Research Ethics Committee under Human Fertility and Embryology Authority research licence R0026. Fresh oocytes and embryos surplus to the clinical IVF treatment programme were obtained from Saint Mary’s Hospital Manchester, graded and prepared as described in Shaw et al 2013 [12] (Fig. 1a).

### Embryo sample preparation and microarray analysis of transcriptome

Upon donation to research, embryos were cultured in G1 medium (Vitrolife, Goteborg, Sweden) covered with a thin layer of mineral oil (Ovoil, Origio, Malov, Denmark) until day 3, followed by G2 medium (Vitrolife) from day 3 to blastocyst at 37 °C, 6% CO_2_ in a humidified atmosphere. Oocytes and embryos at a range of developmental stages, including four oocytes, four 4-cell embryos, three 8-cell embryos, 32 individual blastomeres obtained from four eight cell embryos (blastomeres) and four blastocysts were lysed and reverse transcribed as previously described [8, 13] and cDNA was prepared by polyA-PCR amplification [14]. Embryos were only used if they both reached the appropriate development stage for time in culture and were morphologically normal. Of the 32 8-cell blastomeres, 8 disaggregated from a single 8-cell stage embryo were used to generate single cell transcriptomic data, the remaining 24 disaggregated from 3 complete sets of 8 individual blastomeres were used for qPCR validation. 8-cell stage embryos used for blastomere isolation were equivalent in stage and appearance to those used for single embryo analysis and, to further reduce bias, we analysed only blastomere sets in which we recovered all 8 blastomeres from an 8-cell embryo. For blastomere disaggregation, whole embryos were exposed to Acid Tyrode’s Solution (Sigma) for ~ 3 s intervals until zona breakdown occurred and individual blastomeres were isolated via mechanical disaggregation. The polyA-PCR technique amplifies all polyadenylated RNA in a given sample, preserving the relative abundance in the original sample [15, 16]. A second round of amplification using EpiAmp (Epistem, Manchester, UK) and biotin-16-dUTP labelling using EpiLabel (Epistem) was performed at the Paterson Cancer Research Institute Microarray facility, as previously described [12]. For each sample, our minimum inclusion criterion was the expression of β-actin evaluated by gene specific PCR. Labelled PolyA-cDNA was hybridised to the GeneChip® Human Genome U133 Plus 2.0 Array (Affymetrix, SantaClara, CA, USA) and data initially visualised using MIAMIVICE software [12]. The statistical and graphical R computing language was used together with Bioconductor packages [17, 18], to assess quality control of microarray data (Array Quality Metrics package) [19] (Additional file 1: Supplementary Methods and Additional file 2: Figure S1) and normalisation undertaken using the Mas5 algorithm [20]. Principal component analysis (PCA) was conducted using Partek Genomics Suite software (St. Louis, MO, USA), with IsoMap cross-validation undertaken using Qlucore Omics Explorer 2.3 (Qlucore, Lund, Sweden).

Differential gene expression was determined using ANOVA (Fig. 1b). Benjamini-Hochberg false discovery rate (FDR) corrected *p*-values ≤0.05 were considered differentially expressed [21]. Genes were defined as expressed at a particular developmental stage if present in at least 75% (or two of three) samples (Additional file 3: Table S1). Normalised eukaryotic initiation factor (EIF) family member expression levels were extracted from the arrays for relative expression across development.

### Quantitative PCR

Gene specific PCR amplification of a panel of pluripotency, polarity and hippo-signalling genes was assessed by qPCR using Power SYBR Green Master Mix (Applied Biosystems) according to the manufacturer’s instructions. Primers were designed to produce a product between 50 and 200 base pairs within the first 500 base pairs of the gene to prevent amplification bias. Each 10 μl reaction contained 2 ng polyA-cDNA template and the cycling was performed in an ABI 7300 real-time PCR system. A 10 min denaturation step at 95 °C was followed by 39 PCR cycles comprising 30s denaturation at 95 °C, 30s annealing at 60 °C and 35 s extension at 72 °C. An extended annealing step of 10 min at 72 °C finalised the run. Identification of a single peak at melt curve analysis signified gene expression. Reactions were performed in triplicate for each individual sample. ΔCt was calculated as 40-Ct value for data presentation purposes. Some samples showed low levels of gene expression however genes were not classed as ‘expressed’ above background level unless detected with 37 or fewer cycles of real-time PCR. We only included a sample in the analysis data set if greater than 3 target genes were defined as expressed. Associations between expressed genes were analysed for significance using a chi squared test. Genes displaying *p* ≤ 0.05 were defined as differentially regulated.

### Networks of genes associated with embryonic development

We generated human protein-protein interaction (PPI) networks based on differentially expressed genes in Cytoscape [22] by inference of protein: protein interactions using the BioGrid database [23] with the BioGrid Plugin for Cytoscape [24]. This network generation approach uses a process of inference where a minimum number of connecting proteins are added between proteins with differential gene expression to form a fully connected PPI network model. As a result we capture a section of the human interactome that is associated with transcriptomic differences. Networks enriched for differentially expressed genes were constructed for each developmental stage. The Cytoscape plugin Moduland [25] was used to identify overlapping modules of protein:protein interactions within each developmental network and modular hierarchy was determined using the centrality score (a quantitative measurement of proximity to the centre of the network – the more central a protein is the more it can influence the effect of all other proteins in the network).

Epigenetic regulator genes were compiled from the Qiagen Epigenetic Chromatin Remodelling Factors PCR array (Qiagen) and the Epigenetic Modification Enzymes PCR array (Qiagen), supplemented with genes identified by the network analysis with a known epigenetic role. A hypergeometric distribution was applied to assess enrichment of individual 8-cell blastomeres for chromatin modification enzymes/remodelling factors. Causal Networks (Z-scores >І2І) were used to assess regulatory elements within the networks [26] (Ingenuity Pathway Analysis [IPA], Qiagen).

### Gene expression barcoding of individual 8-cell blastomeres

Frozen robust multi-array analysis (fRMA) [27] was used to define absolute expression by comparison to publically available microarray datasets within R and an expression barcode was defined for each specific tissue and cell type [28, 29]. This GeneBarcode was used to generate single sample networks for the individual blastomeres. Using GeneBarcode, we considered transcripts expressed in 2–5 blastomeres, resulting in 5044 genes for downstream analysis.

### Change point analysis of developmental gene expression

Our embryonic development transcriptomic data (Oocyte, 4-cell, 8-cell and Blastocyst) were filtered by using PCA analysis (Qlucore Omics Explorer 2.3), and 1676 probes were selected with a 1.2 cut-off for the projection score [30]. Then change point analysis was performed in order to identify times in embryonic development when there was a distinct shift in the distribution of gene expression, using the “changepoint” package [31] within R [17]. The transcript expression levels across the stages between two change points were replaced by the average value within this interval and the transcripts were then clustered using hierarchical clustering.

### RNAseq analysis

We analysed previously published single-cell RNAseq data from 81 individual 8-cell human blastomeres [32]. Transcripts per million (TPM) expression values were visualised in Qlucore Omics Explorer 2.3 and outliers were removed. These were normalised for inter-individual variation (mean centred by the embryo donor couple) and by application of a projection score [30] of 0.21 to remove noise (*n* = 59). Gene sets identified were analysed for Gene Ontology and Canonical pathway enrichment using DAVID [33] and mapped onto our differentially regulated 8-cell blastomere network modules.

## Results

### Hierarchy of network modules across embryo development

**Fig. 2:**
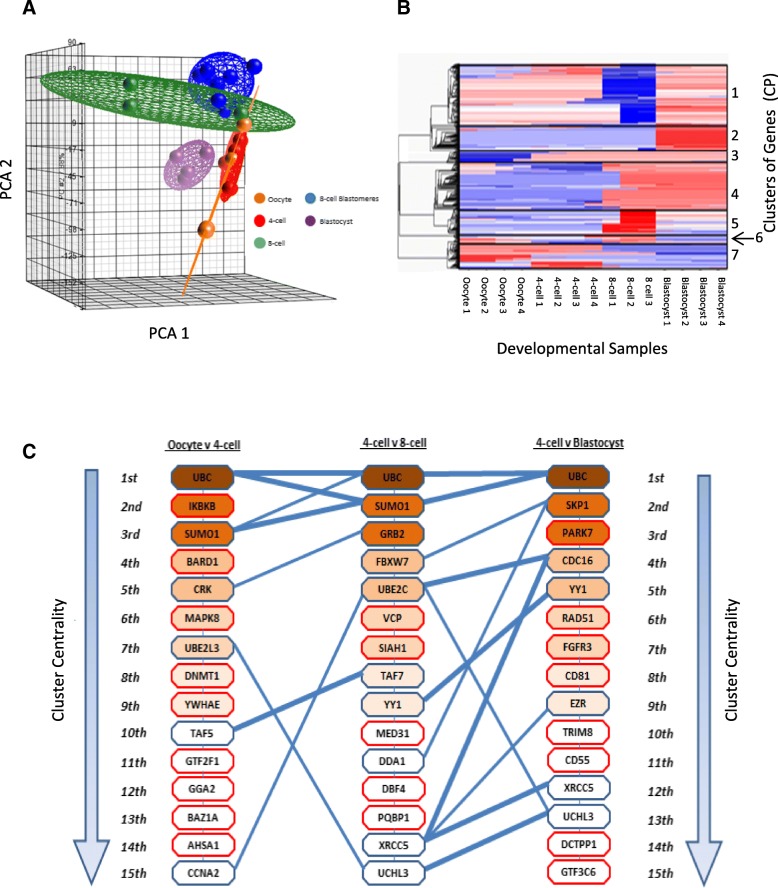
Embryonic transcriptomic profiles. **a**) Principle Component Analysis (PCA) of Mas5 normalised expression values across the human embryonic developmental stages, four oocytes (orange), four 4-cell embryos (red), three whole 8-cell embryos (green), eight 8-cell blastomeres (blue) and four blastocysts (purple). Ellipsoids represent variation within a specific developmental stage. **b**) Heat map of change point matrix for developmental series displayed in panel A. Red represents increased expression and blue decreased expression. **c**) Overlap of network modules across human embryo development. The top 15 modules are extracted from each developmental stage networks and listed in hierarchical order from (1st) most central in the network to (15th) less centrally connected in the network. The gene labelled on the module represents the most centrally connected gene within the module. Blue lines indicate modules which have > 2 shared members between the different developmental stages and the thicker the line the greater the number of shared genes. Modules outlined in red have no shared central elements with the following or preceding developmental stage

We carried out single embryo and blastomere polyA-PCR, global transcriptome profiling on replicate oocytes (*n* = 4) and embryos at the 4-cell (n = 4), 8-cell (*n* = 3) and blastocyst (n = 4) stage, together with 8 individual blastomeres disagregated from one 8-cell embryo (Figs. 1, 2a). PCA analysis revealed that the intact 8-cell embryos and the individual 8-cell blastomeres demonstrated overlapping transcriptomes (Fig. 2a), indicating that the embryo disaggregation procedure for isolating blastomeres had not significantly altered the blastomeres’ expression profiles.

Over the developmental series of embryonic stages we identified 7 key transitions in differentially expressed gene probe-sets using change point analysis (Fig. 2b). Genes expressed in the oocyte and 4-cell embryo but not subsequently (Changepoint group 7; CP7) were considered to be maternally expressed only, those expressed in the oocyte and subsequently at 8-cell (CP6), or oocyte/4-cell and subsequently at blastocyst (CP1), were considered to be expressed both maternally and embryonically following EGA, while those not expressed in the oocyte but only at subsequent stages were assumed to reflect EGA at early (CP3), mid (CP4 and CP5) and late (CP2) developmental stages. The gene ontology associated with each change point group was determined (Additional file 3: Table S2).

Gene networks of the transition for each embryonic stage were constructed by inference to the human interactome network (BioGRID version 3.2.110) (Additional file 2: Figure S2A and B) and a hierarchy of gene modules were defined using the topological property of centrality (Additional file 2: Figures S2C and D). Overlapping network modules were identified and centrally connected genes were used to assess the crosstalk between modules (Additional file 2: Figure S2E and F). We tracked centrally connected genes across our human embryo samples, highlighting features which are maintained throughout development or unique to a certain embryonic stage (Fig. 2c).

Modules expressed in embryos post-EGA, or conserved in both the 8-cell and blastocyst, were deemed to reflect transcription following early and/or later EGA in preparation for implantation and continuing development (Fig. 2a). Definition of network modules in the 8-cell embryo and blastocyst (Additional file 2: Figure S2A-D) were used as a basis to identify upstream regulatory elements (Additional file 2: Figure S2E and F). This approach revealed relationships between *TRIM28/KAP1, MDM2, HDAC2* and *TP53* and the networks of genes they regulate to be of central importance during 8-cell to blastocyst development (Additional file 1: Supplementary results, Additional file 3: Table S3, Additional file 2: Figure S3). Network models defined using inference to the human interactome were confirmed and the robustness of the identified gene modules were assessed by comparison to co-expression networks (Additional file 2: Figure S4).

### Mapping TE or ICM gene expression signatures to individual 8-cell blastomeres

**Fig. 3:**
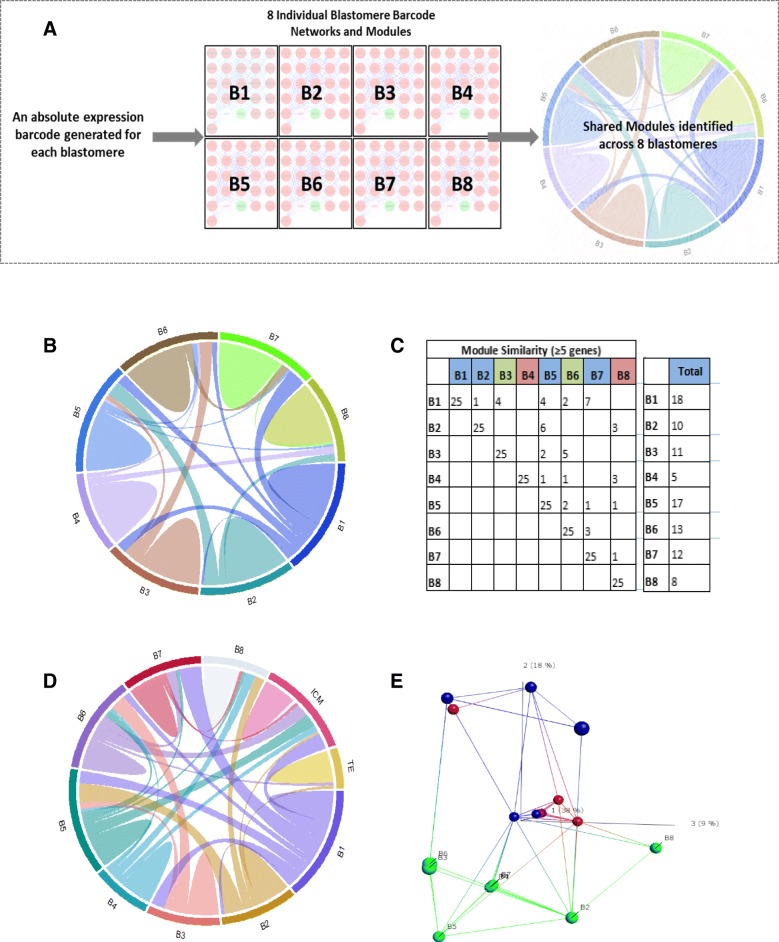
Comparison of individual 8-cell blastomere, inner cell mass (ICM) and trophectoderm (TE) network modules. **a**) Schematic of methodology. Frozen robust multi-array analysis (fRMA) was used to define absolute expression by comparison to publically available microarray datasets within R and an expression barcode was defined for each cell. This GeneBarcode was used to generate single sample networks and identify modules for the individual blastomere samples. Blastomere module similarity was visualised using circos plots. **b**) Individual 8-cell blastomere module similarity. Similar modules between pairs of blastomeres are tabulated and visually represented by connections on circos plots. Similar modules have ≥5 genes. **c**) Tabulated circos plot. Colours of blastomeres represent groups of similarity. The total column shows total overlap in modules for each blastomere, indicating B4 and B8 are the least similar to others. **d**) Individual 8-cell blastomeres, ICM (*n* = 5) and TE (n = 5) module similarity (≥5 genes per module). **e**) Nearest neighbour PCA analysis (isoMap) reveals similarity of individual 8-cell blastomeres (green), TE (red) and ICM (blue)

We defined the transcriptional signatures underlying maintenance of pluripotency and cell lineage separation in the human embryo by comparing the transcriptomes of individual 8-cell blastomeres (B1-B8, Fig. 3). From these data we constructed interaction networks for each blastomere to reveal a hierarchy of modules of highly connected genes (Fig. 3a). We identified upstream regulatory patterns for each blastomere and overlaid the upstream regulatory genes onto blastomere network modules (Additional file 2: Figure S5); the high level of overlap (*p*-value range 0.01–1.2 × 10^− 23^) provided confidence in our gene identifications. Module similarity between the blastomeres was visualised via connectivity in circos plots based on the degree of module overlap (shared ≥5 genes) (Fig. 3b). B4 and B8 were less similar to other blastomeres, whilst B1 shared the greatest amount of similarity to other blastomeres, in particular B3, B5 and B7 (Fig. 3c).

To assess if the differences between the blastomeres represented transcriptional priming towards either ICM or TE lineage, we repeated the same analysis with the inclusion of TE (*n* = 5) and ICM (n = 5) samples (from Stevens et al 2018 [34]) (Fig. 3d). Based on the degree of module overlap (shared ≥5 genes), 5 blastomeres are similar to the ICM, B1, B5 and B6 showed statistically significant overlap (p-value range 8.6 × 10^− 22^ to 2.4 × 10^− 82^). To verify our circos plots, we visualised the blastomere, TE and ICM global expression profiles using IsoMap dimensional scaling (Fig. 3e). These analyses revealed similarities in global gene expression between blastomere B3, B4, B7, B1, B6 and B5 with ICM, and blastomere B8 and B2 with TE, providing independent validation of the overlaps shown in the network derived circos plot and indicating that the TE expression profile was similar to B2, B5 and B8. B2 was therefore identified as TE-like in all three independent analyses, suggesting that blastomere cell fate may be primed at this stage.

### 8-cell blastomeres are not transcriptionally equivalent

In order to explore blastomere cell fate and potential mechanisms in more detail we carried out single embryo and blastomere polyA-PCR and qPCR on a further 24 individual blastomeres disaggregated from 3 complete sets of 8 individual blastomeres. We screened for the expression of genes associated with the Hippo signalling pathway which is involved in specifying TE cell fate [35] (*LATS2, YAP, TEAD4, TAZ, CDX2*), cell polarity [36] (*PARD3, PARD6A, EZRIN, PRICKLE2* and *CDX2*) and cell pluripotency [37] (*SOX2* and *OCT4*) (Additional file 2: Figure S6). No embryo was devoid of *LATS2*, with expression detected within at least 2 of the 8-cell blastomeres. The blastomeres lacking *LATS2* but expressing *TAZ/YAP, TEAD4* and *CDX2* may have greater potential to give rise to future TE. *EZRIN* was the only polarity gene expressed in the majority of 8-cell blastomeres; however levels of expression varied greatly between individual cells. We observed no clustering of gene expression by embryo and the differences in expression of genes involved in hippo signalling, polarity and pluripotency pathways between the individual blastomeres verified the finding from whole transcriptome data that 8-cell blastomeres were not transcriptionally equivalent.

### Expression of eukaryotic initiation factors (EIFs) at the time of EGA

**Fig. 4:**
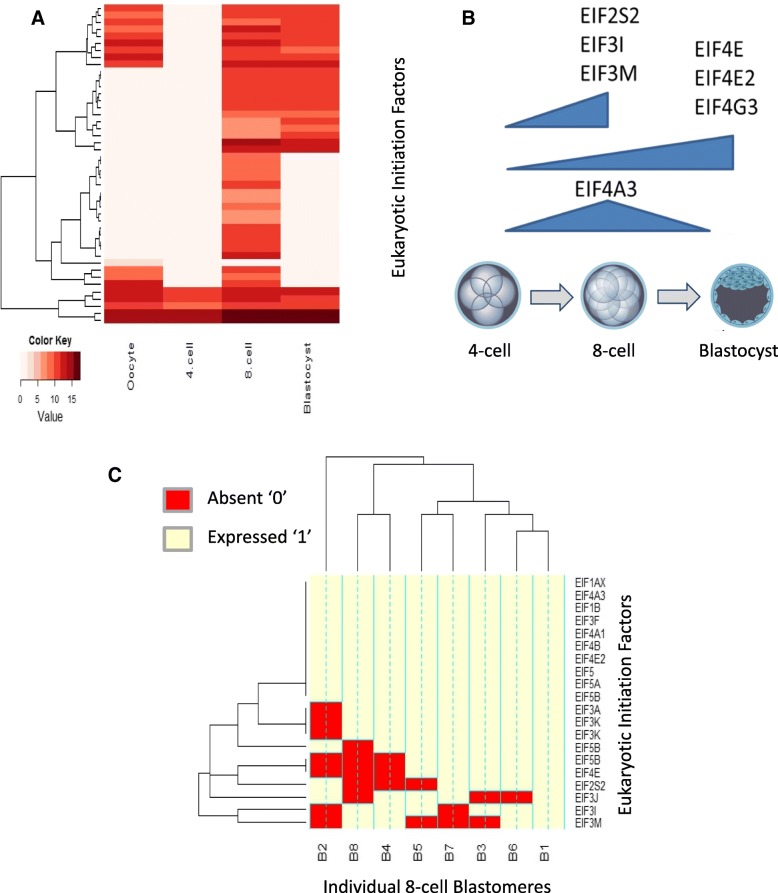
Eukaryotic Initiation Factor (EIF) expression across embryo development and the individual 8-cell blastomeres **a**) Heat Map to represent the 45 EIFs expressed constitutively within at least one oocyte or embryonic stage. Mas5 flags with 75% expression within a specific developmental stage are classed as expressed. **b**) Schematic indicating differentially expressed EIFs (Benjamini-Hochberg FDR ≤ 0.05) between the 4-cell embryo, 8-cell embryo and blastocyst. **c**) Frozen robust multi-array analysis (fRMA) was used to define absolute expression by comparison to publically available microarray datasets within R and an expression barcode was defined for each 8-cell blastomere. The heat map represent EIFs expression within individual 8-cell blastomere expression barcode data, genes with a score of ‘1’ are present and ‘0’ are absent. Half of the EIFs associated with EGA are expressed in all 8 blastomeres, the other half are variably expressed, with blastomere B1 expressing the full complement (10/10), while B2 expressed the fewest (3), with B8 expressing 5/10

Expression and activity of EIFs is critical to successful EGA [38]. Whole transcriptome gene expression of the EIF family was significantly upregulated in the 8-cell and blastocyst (Fig. 4a) and this expression pattern closely followed the general wave of transcripts initiated during EGA [39]. Altogether, 45 EIFs were expressed during pre-implantation development (Fig. 4a). Nineteen EIFs were differentially regulated between the 8-cell embryo and blastocyst; *EIF2S2*, *EIF3I*, and *EIF43M* are up-regulated at the 8-cell, *EIF4E, EIF4E2* and *EIF4G2* are up-regulated in the blastocyst (with *EIF4E, EIFE2* and *EIFG3* regulated by the *TRIM28* network, Additional file 2: Figure S3A), and *EIF4A3* was upregulated in the 8-cell embryo compared to both the 4-cell and blastocyst stage embryo (all FDR modified *p*-value ≤0.05) (Fig. 4b).

The role of EGA in blastomere identity was assessed by examining that transcriptome of individual 8-cell blastomeres to reveal if they displayed varying levels of EIFs. Half of the EIFs differentially regulated in the 8-cell embryo and blastocyst, were present in all individual 8-cell blastomeres, including the 8-cell-specific *EIF4A3*. However the remaining EIFs displayed varying expression levels, with B1 expressing all (10/10) of these EIFs and B2 (3/10) and B8 (5/10) expressing the least number of EIF family members (Fig. 4c).

### Enriched epigenetic signature at the 8-cell stage

As epigenetic modifier genes such as *TRIM28* were differentially expressed during preimplantation development (Additional file 2: Figure S3A), we constructed networks of chromatin modifying enzymes/remodelling factors (Additional file 3: Table S4). More Epigenetic regulatory genes were expressed in the 8-cell embryo (102 genes) compared to the blastocyst (40 genes). Only two genes, *HDAC2* and *YY1*, were upregulated at both stages compared to the 4-cell embryo. Both of these genes were identified as key 8-cell and blastocyst network members (Additional file 2: Figure S2E and F). *HDAC2* is a downstream target of the blastocyst *TRIM28* network (Additional file 2: Figure S3A), whilst *YY1* is a centrally connected gene (Additional file 2: Figure S2C and D) in the 8-cell and blastocyst embryo. Overall, the larger subset of histone acetyltransferases, methyltransferases and deacetylases identified in the 8-cell embryo, indicated that these genes play a part in epigenetic remodelling at this stage.

**Fig. 5:**
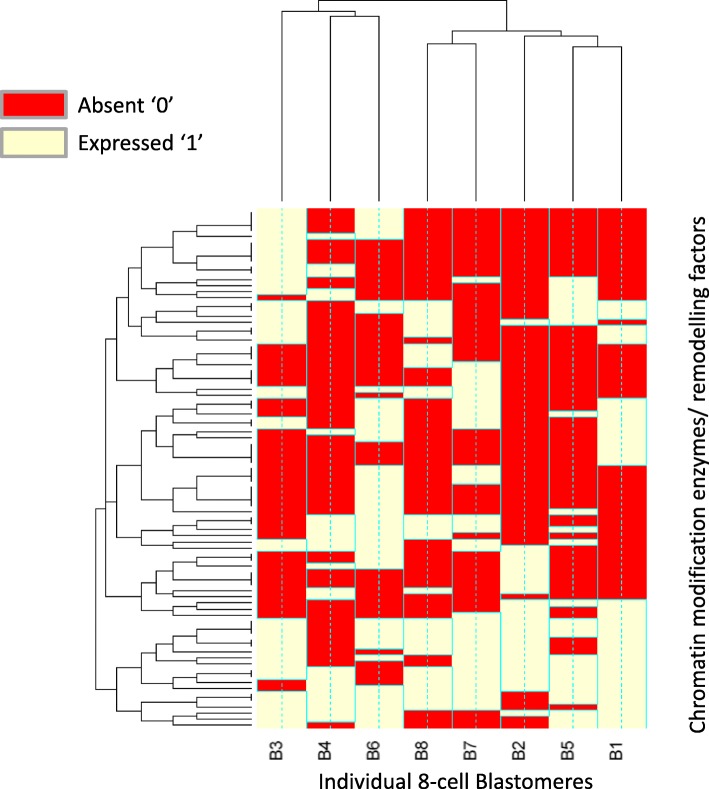
Chromatin modification enzymes/remodelling factors gene expression barcode data within individual 8-cell blastomeres. Frozen robust multi-array analysis (fRMA) was used to define absolute expression by comparison to publically available microarray datasets within R and an expression barcode was defined for each 8-cell blastomere. The heat map represents gene expression within individual 8-cell blastomeres expression barcode data, genes with a score of ‘1’ are present and ‘0’ are absent. The global epigenetic gene expression pattern reveals two groups of individual 8-cell blastomeres; B3/B4/B6 and B1/B2/B5/B7/B8

Due to the upregulated epigenetic-associated gene expression at the 8-cell stage, we assessed the expression of epigenetic regulatory genes within the individual 8-cell blastomeres (Fig. 5). Individual 8-cell blastomeres were significantly enriched (*P* < 3.4 × 10^− 20^) for chromatin modification enzymes and remodelling factors. *TRIM28* network genes, *HDAC2* and *ZSCAN4* were expressed in all blastomeres. However global epigenetic gene expression patterns revealed two groups of individual 8-cell blastomeres; B3/B4/B6, and B1/B2/B5/B7/B8.

### Comparison to published single blastomere RNAseq data

**Fig. 6:**
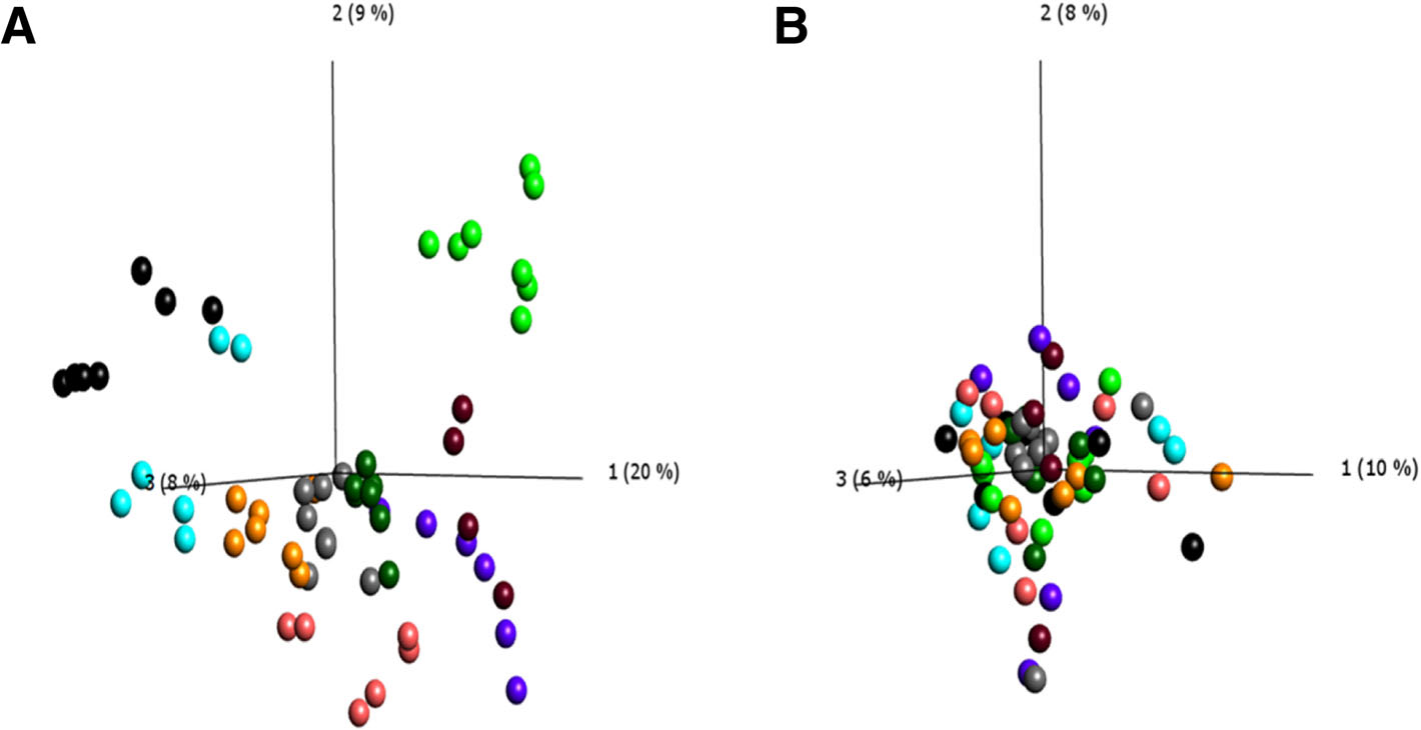
Individual 8 cell blastomere RNA-Seq data (from Petropoulos et al). Transcripts per million (TPM) expression values were visualised using principal component analysis (PCA) and outliers were removed. After outlier removal we used 59 of the 81 published samples. **a**) PCA representing the expression of individual 8-cell blastomeres coloured according to the individual 8-cell embryo of origin. **b**) PCA of 8-cell blastomeres normalised for embryo of origin and variance filtered by application of a projection score of 0.21 to remove noise

To validate and extend our findings of blastomere transcriptional heterogeneity, we analysed single-cell RNAseq data from 81 individual 8-cell human blastomeres [32]. After outlier removal, 59/81 published blastomere datasets of high quality (embryos in which ≥4 of the 8 blastomeres were recovered) were used in further analysis. A PCA of the remaining blastomeres highlighted that the greatest variation in gene expression existed between the individual embryos rather than the individual blastomeres (Fig. 6a). Once samples were normalised for inter-embryo variation we were able to detect differences between individual blastomeres regardless of their embryo origin (Fig. 6b). We identified the presence of 4 sets of genes; of which group 3 was enriched (*p* = 0.0012) for Pluripotency of Embryonic Stem Cells (Additional file 1: Supplementary Results, Additional file 2: Figure S7 and Additional file 3: Table S5).

## Discussion

**Fig. 7:**
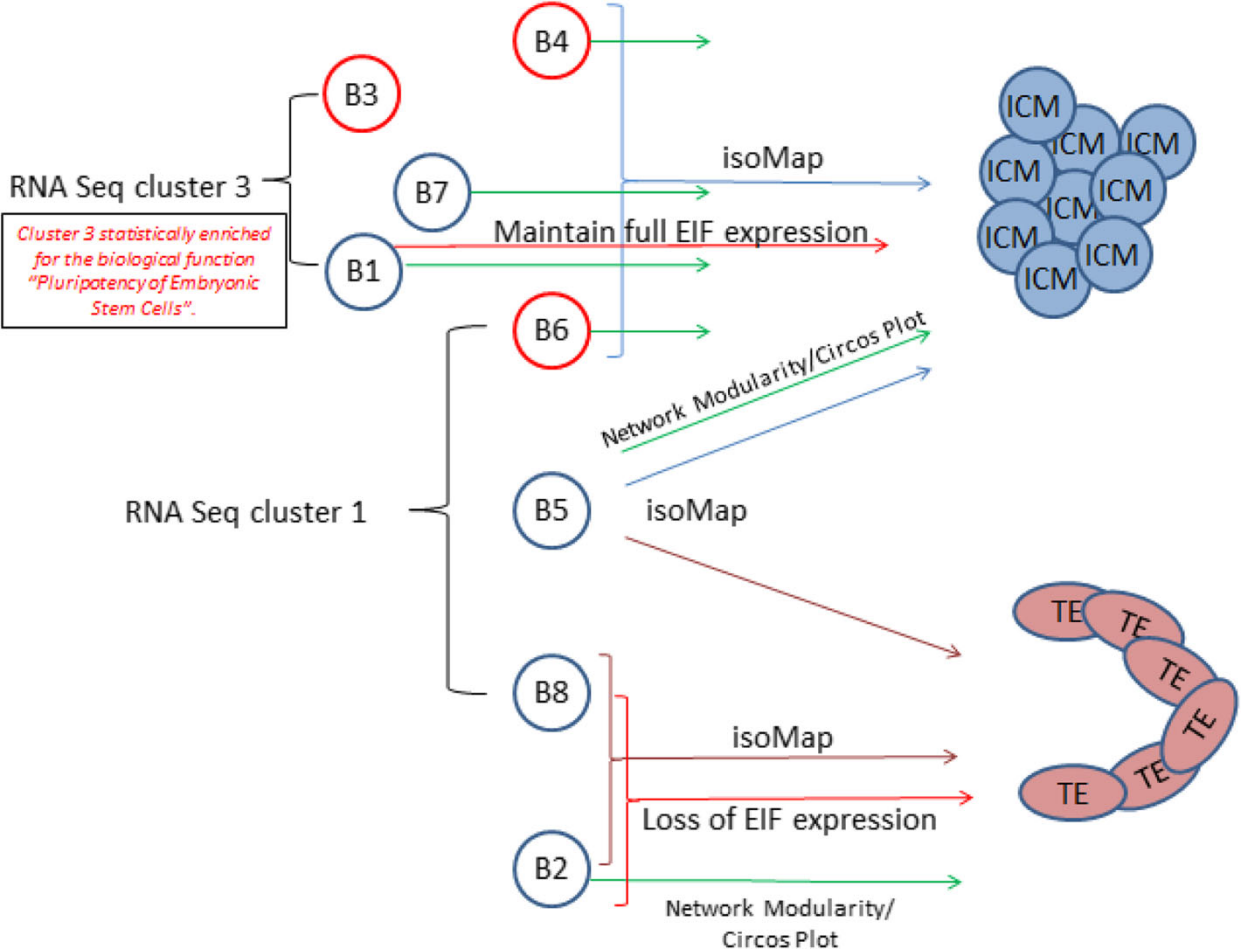
Summary schematic representing 8-cell blastomere genomic divergence. Using a combination of systems biology techniques including network analysis, nearest neighbour PCA (isoMap), EIF expression and epigenetic-associated gene expression we have detected two sets of blastomeres within an 8-cell embryo which exhibit diverging tendencies towards ICM and TE. Nearest neighbour PCA analysis (isoMap) reveals similarities in global gene expression between blastomere 3, 4, 7, 1, 6 and 5 with Inner Cell Mass (Blue brackets/arrows), and blastomere 8 and 2 with Trophectoderm (burgundy brackets/arrows). Green arrows represent similarity in network modules, as highlighted by circos plots. Black bracketed Blastomere 3, 7 and 8 are enriched in RNAseq cluster 3 and Blastomere 6, 5 and 8 are enriched in RNAseq cluster 1. Red brackets/arrows represent blastomeres with the greatest maintenance and loss of developmental EIF expression. B3, B4 and B6 (red coloured blastomeres) have distinct epigenetic associated gene expression signatures from those expressed by blastomeres B1, B2, B5, B7 and B8 (blue blastomeres)

Although there is an increasing body of information about preimplantation human development, relatively little is understood of molecular mechanisms and in particular there is little quantitative information on regulatory gene networks governing the first cell fate decisions in development. Our analysis extends understanding in this area significantly by identifying gene networks involved in EGA and blastomere transcriptional identity and cell fate (Fig. 7).

### Gene networks involved in development during EGA

In humans the major wave of EGA occurs between the 4-cell and 8-cell stage [5, 6], although the mechanisms by which this activation is translated into downstream regulation of embryonic development are unclear. We identified 7 distinct patterns of expression in the human embryonic transcriptome corresponding to different early or late patterns around the time of EGA. Eukaryotic initiation factors (EIFs) are closely associated with transcription activation in mammalian development [40]. We report here a pattern of EIF expression which closely follows EGA, and may be responsible for translating its impact. EIF expression peaked in the 8-cell embryo, which agrees with data of Vasenna et al, who identified three waves of transcriptional activation in humans; the last wave occurring at the 6 to 8-cell stage being responsible for the transcription of the largest number of genes [6]. *EIF2S2, EIF3I*, and *EIF4M* are up-regulated in the 8-cell embryo, with only *EIF2S2* having been previously detected in hESCs and mESCs [41]. EIF expression and activity seems likely to be one of the main mechanisms driving expression of the embryonic developmental programme post-EGA.

Using multiple methods of analysis provides a high confidence framework for understanding the relationship between gene networks at different stages of human preimplantation development. This revealed that *TRIM28/KAP1, MDM2, HDAC2* and *TP53* and the networks of genes they regulate are of central importance at the 8-cell to blastocyst transition. *TRIM28* recruits chromatin modification enzymes and remodelling factors to form a repressive chromatin complex and its expression is essential in mice [42]. We report that *TRIM28* controls a central network of 46 genes, regulating *MYC, TP53* and *MDM2*, suggesting this is essential in embryonic development across species, including humans.

Our analysis showed that genes involved in epigenetic regulation were enriched during late EGA. This expression coincides with the highly transient period of genomic and epigenomic reorganisation [43–46]. We observed the greatest epigenetic signature enrichment in the 8-cell embryo, with more histone deacetylases, methyltransferase and demethylases expressed than DNA methyltransferases and demethylases. DNA modifications are thought to be the main form of epigenetic regulation applied later in development when cells differentiate into particular cell types and genes exhibit long term repression, whereas histone modifications are the major form of gene silencing in early differentiation [45]. The removal of histone modifications is enzymatically easy and so may be preferable during dynamic periods of genome reorganisation, whereas removal of such groups from DNA carry the risk of deleterious DNA mutations. Our analysis of transcriptomic data suggest that in the preimplantation human embryo histone modifications may be the main epigenetic regulatory mechanism during the transient and dynamic period of genome reorganisation. Although, of course, protein synthesis and activity of epigenetic regulators may not necessarily follow transcript expression and further epigenomic analysis would be required to confirm this observation. However, modifications to the epigenome are closely correlated with the transcriptome in developing systems [47]. Epigenetic regulatory networks and chromatin modifying genes in particular were expressed at the 8-cell stage, and in light of their potential role in gene silencing, we explored their association with blastomere identity. Epigenetic gene expression patterns revealed two groups of 8-cell blastomeres (Fig. 7).

### Networks involved in 8 cell blastomere identity and fate

To understand the molecular mechanisms by which individual 8-cell blastomeres achieve diverging cellular identities and either retain pluripotency or differentiate towards TE, we assessed the transcriptome of individual 8-cell stage blastomeres using gene network analyses. It has been previously shown that blastomeres have different transcriptional [48–50] and epigenomic identities [51]. In this manuscript we have defined the relationship with respect to both the global transcriptome and to gene families involved in specifying cell fate. Analysis of genes involved in the hippo signalling pathway, cell polarity and pluripotency showed that no individual blastomere exhibited an identical expression profile to any other blastomere within the same embryo or to those of other 8-cell embryos. The generation of single sample networks for the individual blastomeres global transcriptomes allowed us to quantify the differences/similarities in modules between blastomeres and to map these with high confidence to the embryonic developmental interactomes identified earlier. Groups of blastomeres were identified as more or less similar to each other, and to global transcriptomes of ICM and/or TE which may represent transcriptional priming towards one or other lineage (Fig. 7).

We examined if the developmental changes identified around the time of EGA mapped onto the blastomere transcriptome, i.e. is blastomere transcriptional identity driven by EGA? The EIF family genes involved in translating EGA expression, revealed a close relationship with blastomere identity. Half of the post-EGA differentially expressed EIFs displayed disparate expression, with blastomere B1 expressing all EIFs and blastomere B2 and B8 expressing the least EIFs, suggesting that loss of EIFs may be a potential first point of differentiation [9, 12].

We accounted for more variation in the data by the application of dimensionality reduction techniques (PCA and IsoMap), which demonstrated a direct relationship between the global transcriptomic profiles of six blastomeres with ICM, of which all but B3 show differentially regulated network module similarities to ICM (Fig. 7). IsoMap also identified a direct relationship in the global genetic profiles of blastomeres B8 and B2 with TE, and blastomere B2 showed differentially regulated network module similarities to TE.

The network approaches used in this work are resistant to random error due to the scale free nature of the interactome [52] and therefore form a good basis for the comparison of blastomeres where inter individual variation has been recognised. A similar separation of the blastomeres is apparent after comparison to the most detailed publicly available RNAseq dataset from Petropoulos et al [32]. Overall, the blastomeres enriched in the Petropoulos RNAseq group 3 genes have a stronger divergence towards ICM, whereas the blastomeres enriched in RNAseq group 1 genes have no particular lineage bias (Fig. 7). Having established the likely transcriptional bias of blastomeres towards ICM or TE, we further suggest that the pluripotent state leading to ICM is associated with maintenance of EIF expression, and epigenetic status.

### Clinical implications

We have applied a combination of systems biology approaches to identify a high confidence framework of human preimplantation embryonic networks focussed on EGA and its relationship to blastomere cell fate. This will be uniquely valuable for understanding early human development, where classical experimental design cannot be easily applied due to the rare and protected nature of human embryos. Moreover our gene regulatory framework highlights points of susceptibility during human embryo development, particularly around gene networks involved in EGA and blastomere fate. This provides the scientific community with the opportunity to explore the mechanisms underlying early programming of development, long term health e.g. according to the Developmental Origins of Health and Disease (DOHaD), and a baseline of normal development against which ART practitioners can assess the impact of new clinical technologies [53, 54].

## Conclusions

Utilising multiple layers of computational evidence, we have detected two sets of blastomeres within an 8-cell embryo which exhibit diverging tendencies towards ICM and TE (Fig. 7). Overall our results suggest that the majority of blastomeres are still pluripotent and are unlikely to be lineage-specified or committed. This observation agrees with previous studies indicating that 5–8 cell blastomeres showed equal expression in all ICM, stem-ness and TE markers [11], and with RNAseq data, revealing relatively little transcriptional divergence at the 8-cell stage [32]. However we have identified significantly more heterogeneity between blastomeres than has been previously reported and through the application of our network module approach we have detected biologically significant biases with functional relevance which may prime cells for the earliest cell fate determination. Our approach allows a greater depth of predicted functional analysis than other previously used single dimensional approaches.

## Additional files

Additional file 1: Supplemental Methods and results (DOCX 3560 kb)

Additional file 2: **Figure S1.** Un-normalised Affymetrix microarrays A) Boxplots represent summaries of the signal intensity distributions of the arrays. Each box corresponds to one array. B) Boxplot outlier detection was performed by computing the Kolmogorov-Smirnov statistic *K*_*a*_between each array’s distribution and the distribution of the pooled data. None of the samples have medians higher than 1.05, which would represent a low quality array C) Density distributions of the log_2_intensities grouped by the matching type of the probes. The blue line shows a density estimate (smoothed histogram) from intensities of perfect match probes (PM), the grey line, one from the mismatch probes (MM). D) RNA digestion plot. The shown values are computed from the pre-processed data. Each array is represented by a single line. E) MA plots (M = log_2_ (I_1_)-log_2_ (I_2_), A = 1/2(log_2_ (I_1_) + log_2_ (I_2_)), where I_1_ is the intensity of the array studied, and I_2_ is the intensity of a “pseudo”-array that consists of the median across arrays. The mass of the distribution in an MA plot should be concentrated along the M = 0 axis, and there should be no trend in M as a function of A. Shown are first the 4 arrays with the highest values of *D*_*a*_, then the 4 arrays with the lowest values. F) An example of feature intensities representing the arrays’ spatial distributions (M). **Figure S2. **A and B) Embryonic genome activation (EGA) interaction networks, differential regulation within the 8-cell and blastocyst compared to the 4-cell. C and D) Metanodes of the 8-cell and blastocyst compared to the 4-cell, metanodes as defined by the Cytoscape plugin ‘Moduland’, metanodes represent genes most central within each module. Red genes represent up-regulation, green nodes represent down-regulation and pink nodes represent non-differentially regulated genes but baseline expressed direct interaction partners. E and F) Tables of network module members. Metanodes represent the most centrally connected gene within a module alongside the next 10 centrally connected genes within each module. Modules are ranked in order from most to least centrally connected within the specific developmental network. Yellow highlighted genes are also identified as Ingenuity causal network genes. **Figure S3. **A) TRIM28 upstream regulatory network in blastocysts. MDM2 is the only target gene regulated by both upstream regulators MYC and TP53. TRIM28 together with MYC and TP53, may represent the upstream transcriptional control network over the MDM2 module in the blastocyst and provide upstream regulation of epigenetic networks. B) MDM2 is identified as a key module and upstream regulator at the blastocyst stage. MDM2 together with 22 participating regulators, controls the expression of 93 differentially regulated genes within the blastocyst. The participating regulator, transcription factor GATA3, is up-regulated 867-fold in the blastocyst. Pink nodes represent genes identified within module analysis and red nodes represent genes identified within both module and upstream regulatory network analysis. Pathway analysis of the MDM2 module reveals statistically over-represented (*p* ≤ 0.05) Reactome pathways, ordered from 1 to 10 according to their significance *p*-value. The MDM2 module is biologically relevant, with 6/10 of the top MDM2 module genes being members of the hedgehog signalling ‘on state’ pathway. **Figure S4.** A) 107 co-expression functional modules B) The frequency a module of a specific size was detected using co-expression analysis. C) Overlap of the intra-modular hubs between different methods. In order to have enough genes for the comparison between different methods, all the co-expression modules for the robustness evaluation were selected by including more than 5 genes. The diagonal of the table indicates the numbers of the total genes in each method; the lower triangular matrix shows the numbers of overlapping genes between the corresponding two methods; the upper triangular matrix shows the hypergeometric *p*-values for the numbers of overlapping genes. **Figure S5.** Each panel represents a single 8-cell blastomeres top 25 network modules (columns) and the top 10 centrally connected genes within each module. Blastomere networks and modules identified using the absolute expression values of 8-cell blastomeres. Modules are ranked in order from most (left) to least (right) centrally connected within the specific blastomere network. The most centrally connected gene within each module are shown in bold and the remaining genes are ranked from most (top) to least (bottom) centrally connected within a specific module. Blue highlighted genes are also identified as upstream regulatory genes. **Figure S6.** qPCR expression (ΔCt) of Hippo signalling, pluripotency and polarity genes across three sets of 8-cell blastomeres. The first set of blastomers are labelled A1-A8, the second set of blastomeres are labelled B1-B8 and the final set of blastomeres are labelled C1-C8. ΔCt was calculated as 40-Ct. Positive and negative bars represent standard error of the mean. **Figure S7.** Heat map of individual 8-cell blastomere RNAseq data extracted from Petropoulos et al. Heatmap displays individual 8-cell blastomeres on the horizontal axis and genes on the vertical axis. Individual blastomeres are clustered according to gene expression similarity. After outlier removal we used 59 of the 81 published samples. Embryo origin normalised and variance filter applied (0.21) to exclude noise. Resulting in 588 probes, separated into four groups based on hierarchal clustering. Red represents increased gene expression and blue represents decreased gene expression. (PPTX 6500 kb)

Additional file 3: Supplemental Tables (XLSX 274 kb)

## Abbreviations

EGA: Embryonic Genome Activation
EIF: Eukaryotic Initiation Factors
FDR: False Discovery Rate
ICM: Inner Cell Mass
PCA: Principal component analysis
TE: Trophectoderm
TPM: Transcripts per million
