# Supplementary material for: Systems based analysis of human embryos and gene networks involved in cell lineage allocation
Source: BMC Genomics. 2019 Mar 5;20:171. doi: 10.1186/s12864-019-5558-8 (PMC6399968; doi:10.1186/s12864-019-5558-8)
Supplement: Supplementary file 1 — Supplemental Methods and results (DOCX 3560 kb) [file 12864_2019_5558_MOESM1_ESM.docx]

**Supplementary Methods**

**Array Quality Metrics**

The statistical and graphical R computing language was used together with Bioconductor packages [1, 2], to assess quality control of microarray data using the Array Quality Metrics package[3]. The array quality metrics package provides a report with diagnostic plots to assess reproducibility, identify apparent outlier arrays and compute measures of signal-to-noise ratio.

**Construction of co-expression network and extraction of MCL functional modules**

A gene co-expression network was constructed based on the gene expressions across multiple developmental stages (oocyte, 4-cell, 8-cell and blastocyst) and the human PPI network (BioGrid version 3.3.123) [4]. The co-expression between each interacted gene pair in the PPI network was calculated by Pearson Correlation Coefficient (PCC). The significant co-expressed interactions (p-values of PCCs lower than 0.05) were kept as edges in the co-expression network with the PCCs as the weights for the edges. Then, the co-expression network was clustered using Markov Cluster Algorithm (MCL) [5] with default parameters, and the clusters, which have more than 10 genes are selected as co-expression functional modules.

To characterize the co-expression functional modules, each co-expression module is enriched with Gene Ontology (GO) [6] biological processes using the hypergeometric test. The GO annotation was downloaded from http://geneontology.org/ on 05-06-2014. The enriched p-values of GO terms were adjusted by Benjamini-Hochberg (BH) false discovery rate (FDR) correction for multiple testing [7]. GO terms with corrected p-values lower than 0.05 were selected as significant functions for the modules.

The crosstalk between an inter-module gene and a module is defined as the statistical significance of the interactions between the inter-module gene and the genes within the module. The statistic significances evaluated by hypergeometric test with p-value lower than 0.05 are defined as cross-talk. In this analysis, only cross-talk between inter-module hubs and modules were considered. The inter-module hubs were defined as a gene with more than 10 edges in the co-expression network which was located out of all the gene functional modules. If an inter-module hub had cross-talk with more than one module, the hub was defined as a pivot.

**Evaluation of the robustness of the modules and pivot genes**

To evaluate the robustness and the reproducibility of the functional modules, we used three different cut-offs (I=2, 3, 4) for MCL as well as two additional clustering methods, Moduland [8, 9] and Weighted Gene Co-expression Network Analysis (WGCNA) [10] to extract the co-expression modules and identify the pivot genes based on the respective modules.

The Moduland plug-in was applied in Cytoscape on the co-expression network. WGCNA was implemented in R, the gene co-expression clusters were extracted based on gene expression data with the most important cut-off “power” for clustering was selected by soft threshold estimation (power=8). Then the protein-protein interactions were combined with each gene cluster to extract all the connected components which were defined as the co-expression modules.

In order to have enough genes for the comparison between different methods, all the co-expression modules for the robustness evaluation were selected by including more than 5 genes. The hypergeometric test was used to evaluate the significance of the overlaps of module genes and pivot genes between different methods.

**Supplementary results**

**Array Quality Metrics**

Before normalisation, the Array Quality Metric (AQM) report was performed, comprising of ten different quality metrics, where in at least 8 out of the 10 metrics no sample was identified as an outlier. Boxplots represent the signal intensity distributions for each array, which should have similar widths and positions (**Supplementary Figure 1A**), and the outlier detection Kolmogorov-Smirnov statistic Ka confirmed none of the samples were outliers (**Supplementary Figure 1B**). The miss-match (MM)/perfect-match (PM) plot showed the density distributions of intensities grouped by matching probe type. The plots show the correct distributions, with the perfect-match probes binding more efficiently and therefore shifted slightly to the right of the miss-match probes (**Supplementary Figure 1C**). RNA digestion plot shows values computed from the pre-processed data, with each array represented by a single line (**Supplementary Figure 1D**). MA plots for several of the arrays demonstrated a trend in the upper range of M which can indicate a higher background level; this can be addressed by normalisation (**Supplementary Figure 1E**). False colour feature intensities, demonstrated features are distributed randomly and no technical errors such as air bubbles affect the arrays (**Supplementary Figure 1F**). The data were normalized using several different algorithms and the post-normalization AQM reports generated, from which we deemed Mas5 to be the most appropriate normalization technique for the developmental series, oocytes through to blastocyst stage embryos.

**Hierarchy of Network Modules across Embryo Development**

Genes from each of the 7 change point analysis groups were analysed for Gene Ontology and Canonical pathway enrichment; CP1 was highly enriched for essential housekeeping pathways involved in cell adhesion, cell junction processes and extracellular protein assembly while CP2 genes were mainly involved in cellular metabolic processes (**Figure 2B and Supplementary Table 2**), CP4 and CP5 were enriched in mitochondrial processes, mRNAsplicing, autophagy and mitophagy. CP7 genes were enriched in pathways involved in junction adhesion and junction interactions, while CP3 and CP6 genes provided poorer levels of pathway enrichment (**Figure 2B and Supplementary Table 2**).

Overlaping network modules with causal network analysis revealed relationships between *TRIM28/KAP1, MDM2, HDAC2* and TP53. In blastocysts, *TRIM28* was identified as a key transcription factor regulating a network of 46 genes including other master regulators such as *c-MYC* and *TP53*. *TRIM28* was predicted by downstream expression patterns to be inhibited, and since this protein contributes to the generation of repressive epigenetic states, its inhibition is predicted to release transcriptional repression on *c-MYC* and *TP53*. Interestingly, the *TRIM28* causal network regulates the expression of *MDM2, HDAC2, EIF4E, EIFE2 and EIFG3* (**Supplementary Figure 3A**), the same eukaryotic initiation factors (EIFs) identified in the blastocyst by ontology (see Expression of Eukaryotic Initiation Factors (EIFs) at the time of EGA, main manuscript). *MDM2* regulated 93 further differentially expressed genes. *HDAC2* displayed the highest expression of all HDACs in our dataset and was the only HDAC up-regulated in both the 8-cell embryo and blastocyst in our two independent analyses. The expression of key *TRIM28* causal network members was validated in two additional single human embryo microarray datasets, a publicly available study [11] and an additional set of ten blastocysts (Helen Smith, unpublished data) (**Supplementary Results Figure 1**)**.** *TRIM28* was shown to be positively expressed in all stages of pre-implantation embryonic development, whilst *c-MYC* was expressed only in the 8-cell embryo and blastocyst.

**Mapping developmental transitions in gene expression to blastomere identity**

We have established that important changes in gene networks take place in time and also in space (the diverging transcriptional identities of 8-cell blastomeres) as a result of EGA. We next sought to determine whether developmental changes driven by EGA map directly onto the blastomeres, i.e. is blastomere transcriptional identity driven by EGA?

We mapped the 7 groups of change point genes (**Figure 2B**) onto our 8-cell blastomere network modules (**Supplementary Results Figure 2**). Maternally expressed-only genes (CP7) did not map strongly to any 8-cell blastomeres, as expected, however maternal genes which were then re-expressed following EGA fell into 2 distinct subgroups, those which also did not map to blastomeres (CP6), and those which despite only weak expression at the 8 cell stage, showed clear mapping to blastomeres (CP1). CP 2, 3, 4 and 5 genes were activated exclusively following EGA, with early EGA (CP3) mapping most strongly and mid EGA (CP4 and 5) and late EGA (CP2) mapping less strongly to blastomeres. B5 mapped to all 3 CP groups which are not expressed at the 8-cell stage, and not to the other 4 CP groups. Conversely, B8 maps to CP groups expressed at 8-cell, with B6 mapping to no CP group (**Supplementary Results Figure 2**).

**Co-expression functional modules**

In order to provide another independent approach to our analysis, we constructed gene co-expression networks. These were constructed from genes sharing similar expression profiles. It has been suggested that common transcriptional regulators may control co-expressed gene clusters (12). Our gene co-expression network contains 8127 nodes (genes) and 22510 edges (co-expressed interactions). The distribution of the degrees (the numbers of the edges connected to the nodes) follows the power-law distribution with the estimated γ=2.73, which suggests that the co-expression network is a scale free network whose degrees follow a power-law distribution with the parameter γ in the range 2<γ<3 [12-14]. One hundred and seven co-expression modules containing more than 10 genes each were extracted from the co-expression network using the MCL clustering algorithm (**Supplementary Figure 4A**). The biggest module includes 114 genes (**Supplementary Figure 4B**). Thirty two out of the 107 modules are significantly enriched in biological processes defined by gene ontology (**Supplementary Results Table 1**). Comparing intra-modular hubs using our two approaches of protein-protein interaction networks and co-expression networks revealed statistically significant levels of overlap (**Supplementary Figure 4C**), providing independent confirmation that we have detected unbiased modules.

**Comparison to published single blastomere RNA Seq data**

Once samples were normalised for inter-embryo variation we were able to detect differences between individual blastomeres regardless of their embryo of origin (**Figure 6B**). After variance filtering to remove noise, 588 probes were visualised on a heat map, resulting in the detection of four sets of genes (**Supplementary Figure 7 and Supplementary Table 5**). We classified gene set 2 as noise due to the poor resolution of the cluster within the dendrogram. We then mapped the three groups of genes onto our 8-cell blastomere network modules (**Supplementary Figure 7 and Supplementary Results Figure 3**) and revealed enrichment for individual blastomere network models with genes from either RNASeq cluster 1 or 3. Blastomeres 1, 3 and 7 were enriched for cluster 3, whereas blastomeres 5, 6 and 8 were enriched for cluster 1. Both sets of genes were enriched for a wide range of general biological processes; however RNA Seq group 3 was uniquely and significantly enriched (p-value= 0.0012) for Pluripotency of Embryonic Stem Cells. Blastomere 2 and 4 showed no bias towards any RNA Seq gene cluster (**Supplementary Figure 7 and Supplementary Results Figure 4**).

1. RCoreTeam: **R: A language and environment for statistical computing.** *R Foundation for Statistical Computing, Vienna, Austria* 2016, [**https://www.R-project.org/**](https://www.R-project.org/).

2. Gentleman RC, Carey VJ, Bates DM, Bolstad B, Dettling M, Dudoit S, Ellis B, Gautier L, Ge Y, Gentry J, et al: **Bioconductor: open software development for computational biology and bioinformatics.** *Genome Biol* 2004, **5:**R80.

3. Kauffmann A, Gentleman R, Huber W: **arrayQualityMetrics--a bioconductor package for quality assessment of microarray data.** *Bioinformatics* 2009, **25:**415-416.

4. Chatr-Aryamontri A, Breitkreutz BJ, Oughtred R, Boucher L, Heinicke S, Chen D, Stark C, Breitkreutz A, Kolas N, O'Donnell L, et al: **The BioGRID interaction database: 2015 update.** *Nucleic Acids Res* 2015, **43:**D470-478.

5. Enright AJ, Van Dongen S, Ouzounis CA: **An efficient algorithm for large-scale detection of protein families.** *Nucleic Acids Res* 2002, **30:**1575-1584.

6. Ashburner M, Ball CA, Blake JA, Botstein D, Butler H, Cherry JM, Davis AP, Dolinski K, Dwight SS, Eppig JT, et al: **Gene ontology: tool for the unification of biology. The Gene Ontology Consortium.** *Nat Genet* 2000, **25:**25-29.

7. Benjamini Y, Drai D, Elmer G, Kafkafi N, Golani I: **Controlling the false discovery rate in behavior genetics research.** *Behav Brain Res* 2001, **125:**279-284.

8. Kovacs IA, Palotai R, Szalay MS, Csermely P: **Community landscapes: an integrative approach to determine overlapping network module hierarchy, identify key nodes and predict network dynamics.** *PLoS One* 2010, **5**.

9. Szalay-Beko M, Palotai R, Szappanos B, Kovacs IA, Papp B, Csermely P: **ModuLand plug-in for Cytoscape: determination of hierarchical layers of overlapping network modules and community centrality.** *Bioinformatics* 2012, **28:**2202-2204.

10. Langfelder P, Horvath S: **WGCNA: an R package for weighted correlation network analysis.** *BMC Bioinformatics* 2008, **9:**559.

11. Xie D, Chen CC, Ptaszek LM, Xiao S, Cao X, Fang F, Ng HH, Lewin HA, Cowan C, Zhong S: **Rewirable gene regulatory networks in the preimplantation embryonic development of three mammalian species.** *Genome Res* 2010, **20:**804-815.

12. Barabasi AL: **Scale-free networks: a decade and beyond.** *Science* 2009, **325:**412-413.

13. Barabasi AL, Bonabeau E: **Scale-free networks.** *Sci Am* 2003, **288:**60-69.

14. Barabasi AL, Oltvai ZN: **Network biology: understanding the cell's functional organization.** *Nat Rev Genet* 2004, **5:**101-113.


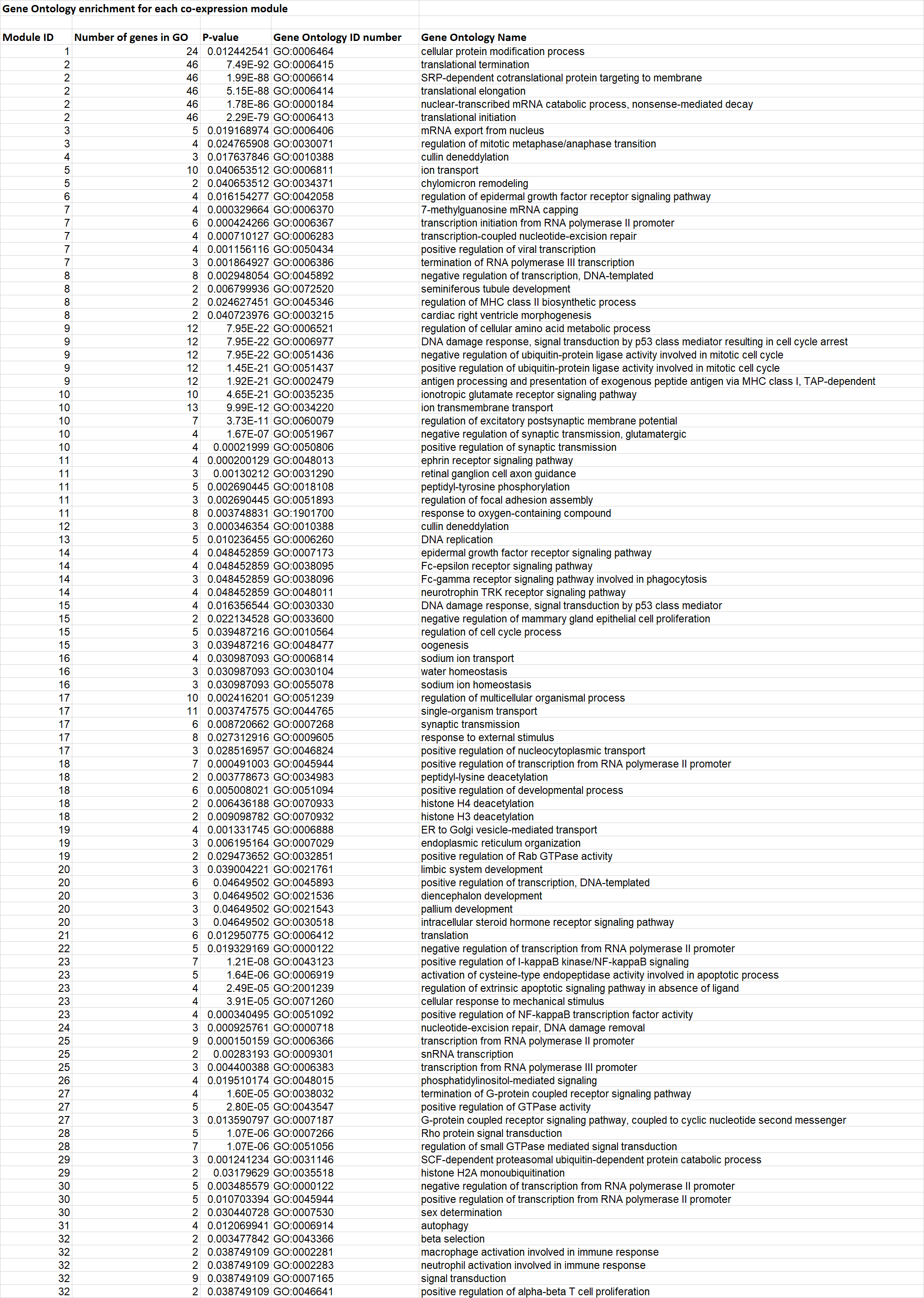


**Supplementary Results Table 1.** Statistically over-represented (p<0.05) Gene Ontology (GO) IDs for given co-expression modules. Modules 1-32 identified, sorted by module ID and the numbers of genes within that modules hitting a specific GO is given in column 2.


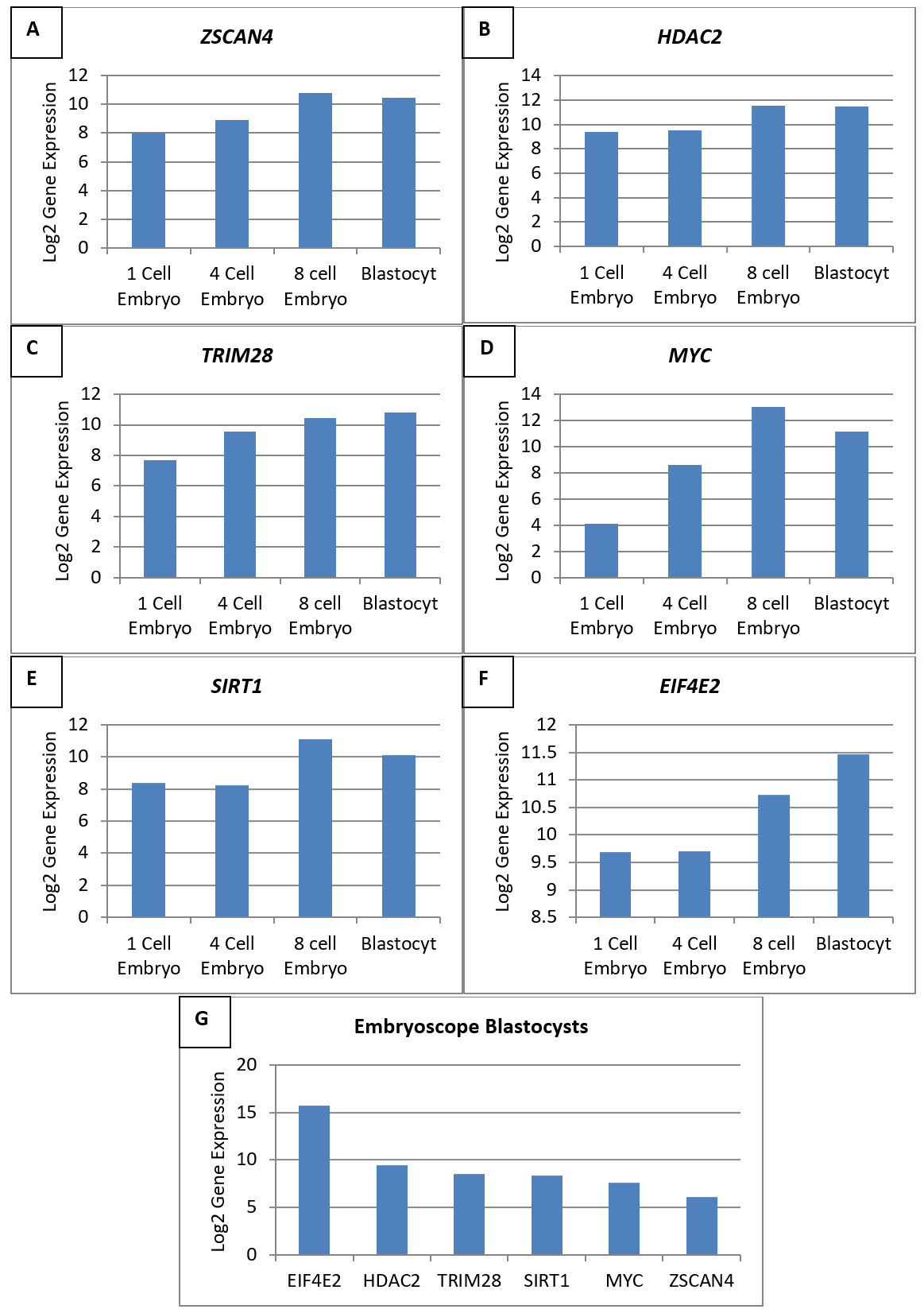
**Supplementary Results Figure 1.** Average probes Mas5 normalised Log2 expression values, validating the expression of key genes identified throughout the pre-implantation embryo. (**A-F**) Expression values around or less than 6 are not expressed. **G**) Average probes Mas5 normalised expressions derived from an additional set of blastocysts (n =10) (Helen Smith, unpublished data).


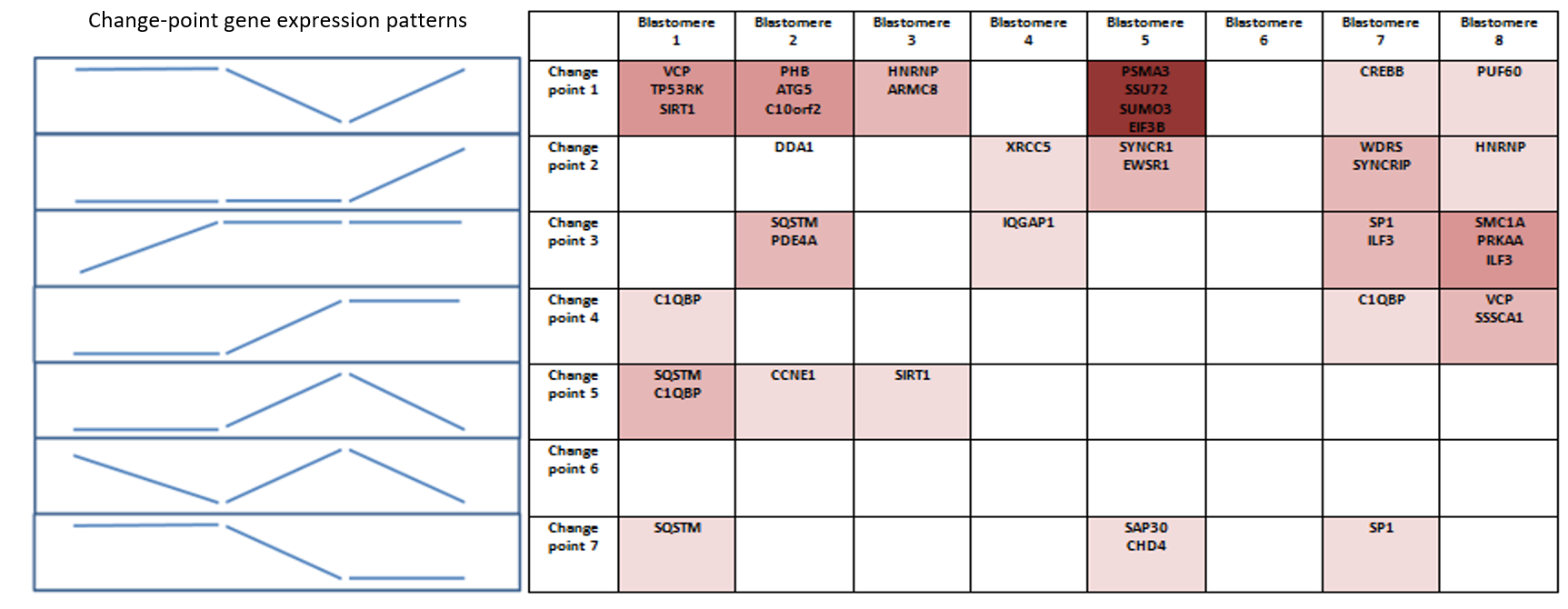


**Supplementary Results Figure 2.** Alluvial diagram representing change-point gene set expression patterns across development and table representing the number of genes from each change point gene set present in the individual 8-cell blastomeres key modules. Blastomere networks and modules identified using the absolute expression values of 8-cell blastomeres.


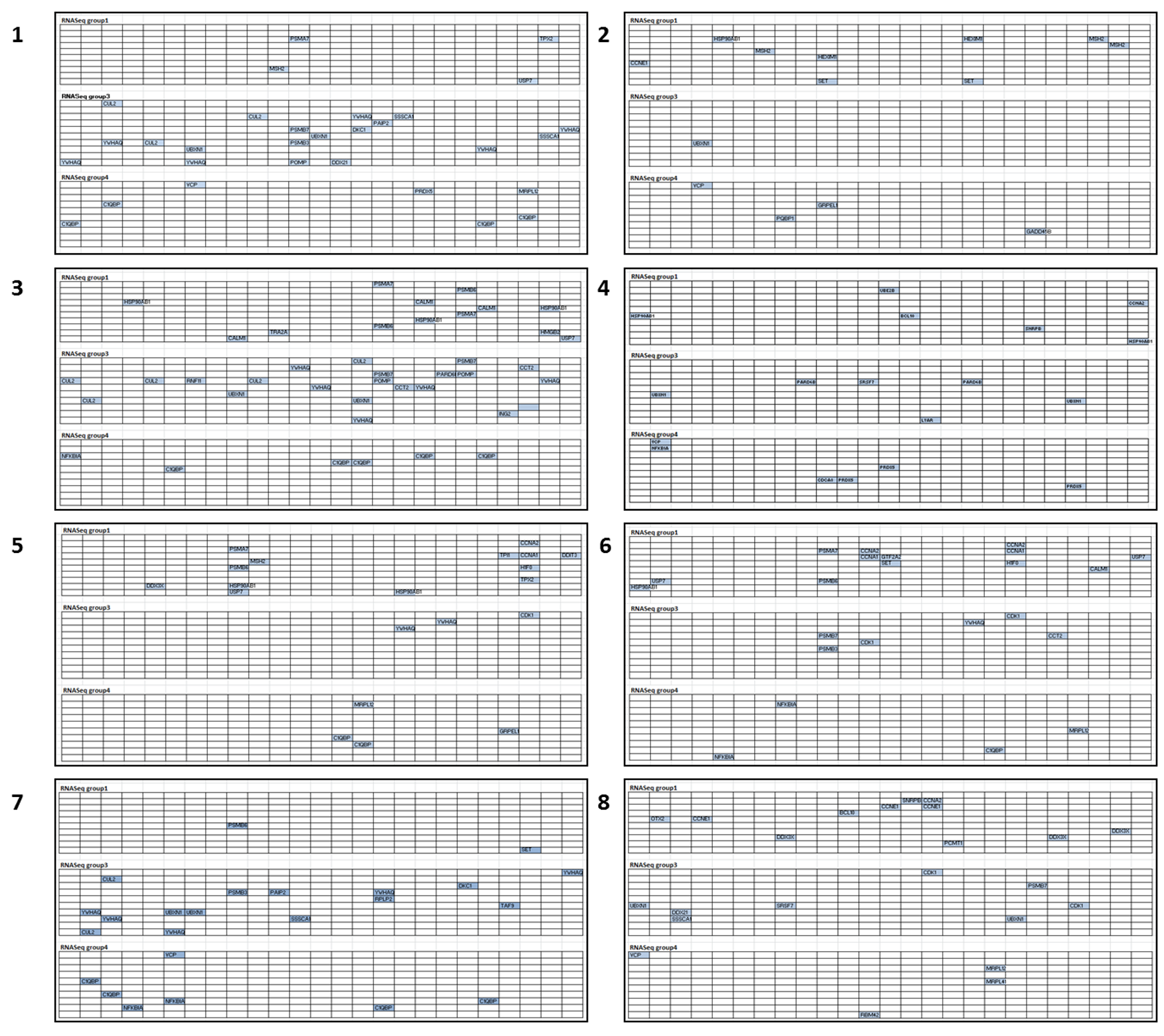


**Supplementary Results Figure 3.** Panels labelled 1-8 represent the individual 8-cell blastomeres hierarchy of top 25 network modules (columns) and the top 10 centrally connected genes within each module. Each individual blastomeres modules are ranked in order from most (left) to least (right) centrally connected The three panels within each blastomere represent genes present in the RNA Seq group 1, 2 and 4 identified from in the Heat map using data extracted from Petropoulos *et al* (Supplementary Figure 7). Genes present within the groups identified by RNA Seq have been overlaid onto the individual 8-cell network modules.


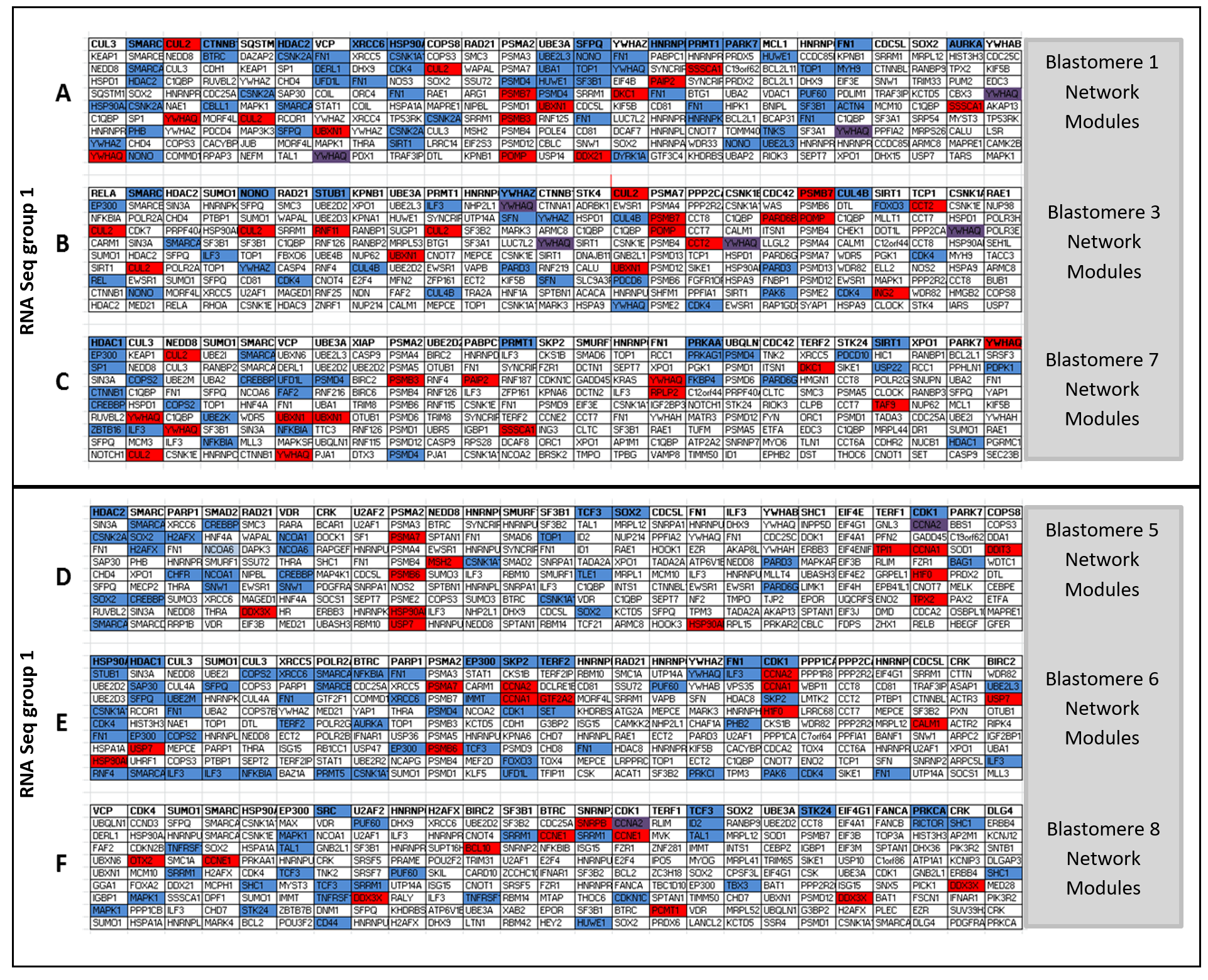


**Supplementary Results Figure 4.** Panels represent blastomere network modules as shown in supplementary figure 5. Two groups of blastomeres are enriched in either RNA Seq cluster 3 genes (A-C) or RNA Seq cluster 1 genes (D-F). RNA Seq groups identified from the Heat map using data extracted from Petropoulos *et al* (Supplementary Figure 7). Modules are ranked in order from most (left) to least (right) centrally connected within the specific blastomere network. The most centrally connected gene within each module are shown in bold and the remaining genes are ranked from most (top) to least (bottom) centrally connected within a specific module. Blue highlighted genes are also identified as up-stream regulatory genes, red genes are identified within the specific RNA Seq cluster and purple are in both.
