# Supplementary figures and images for: Systems based analysis of human embryos and gene networks involved in cell lineage allocation
Source: BMC Genomics. 2019 Mar 5;20:171. doi: 10.1186/s12864-019-5558-8 (PMC6399968; doi:10.1186/s12864-019-5558-8)

## Slide 1
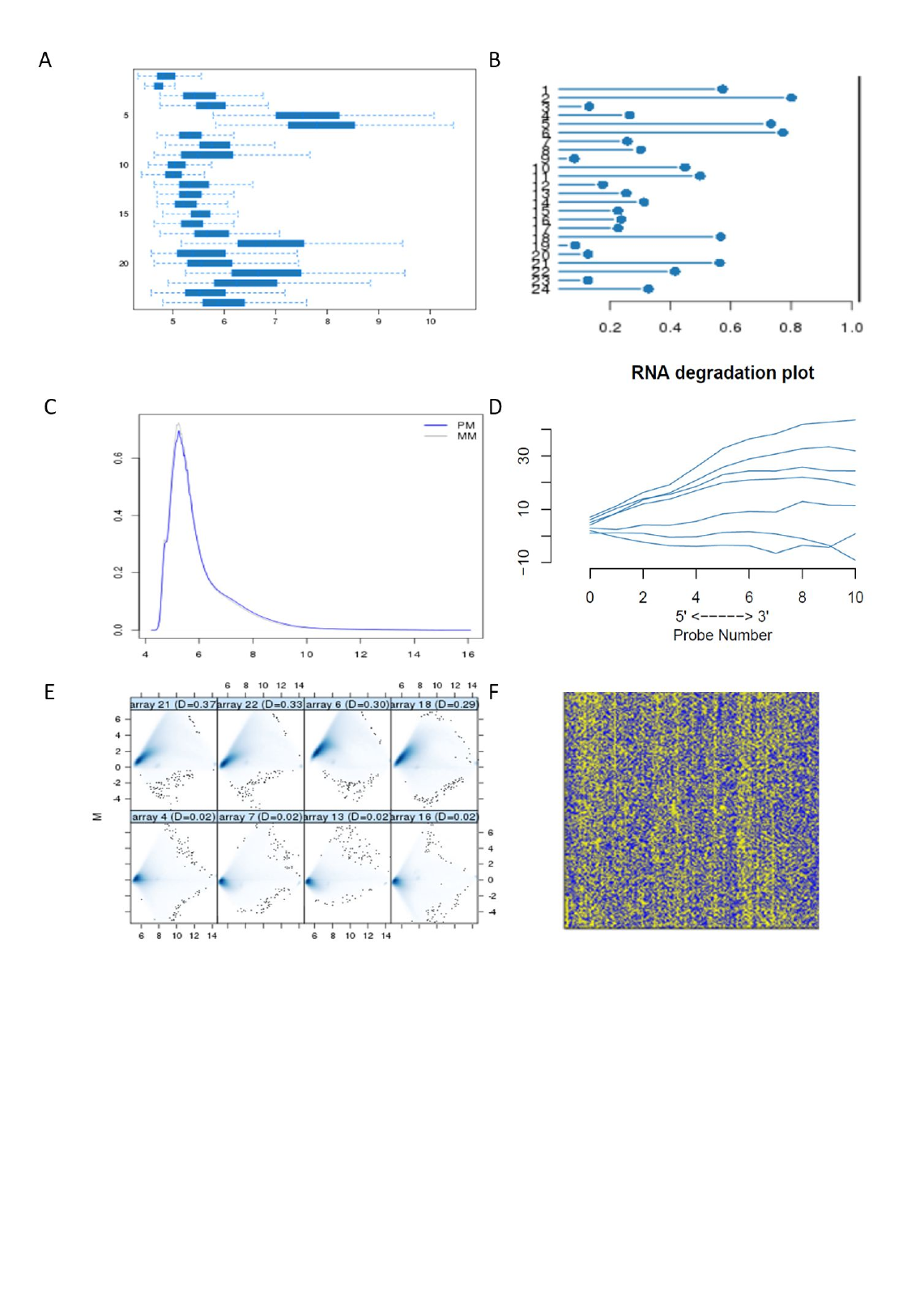

## Slide 2
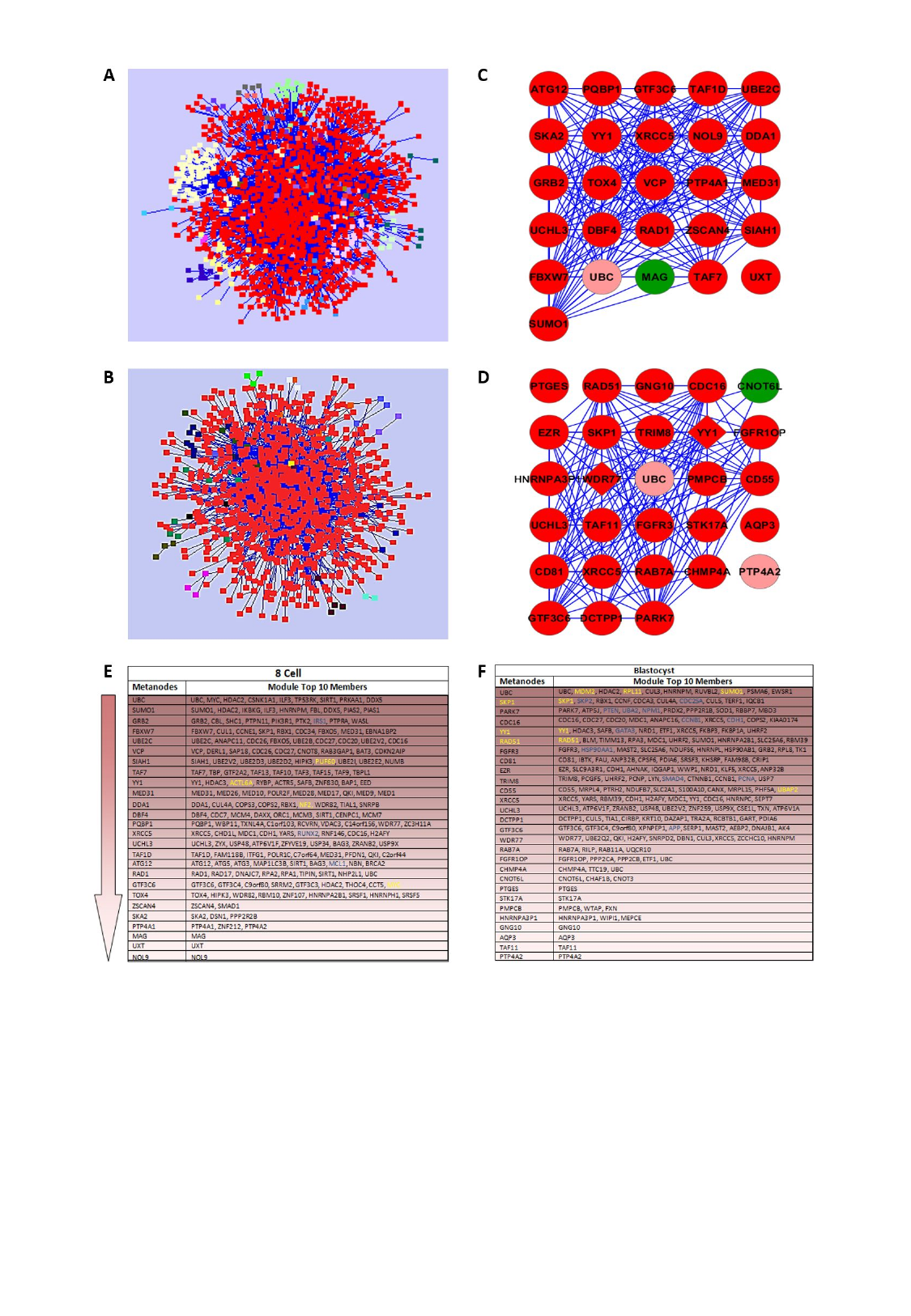

## Slide 3
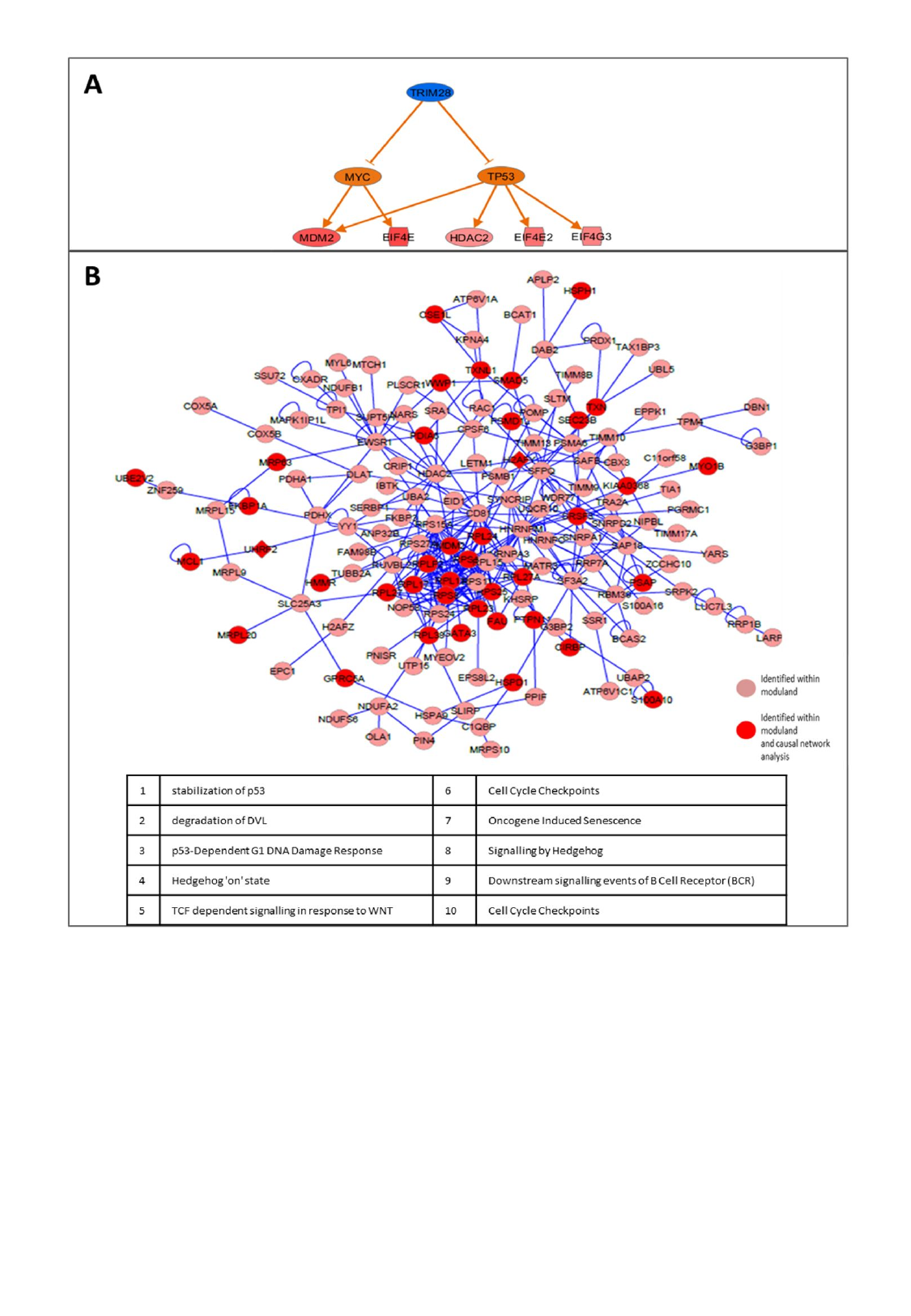

## Slide 4
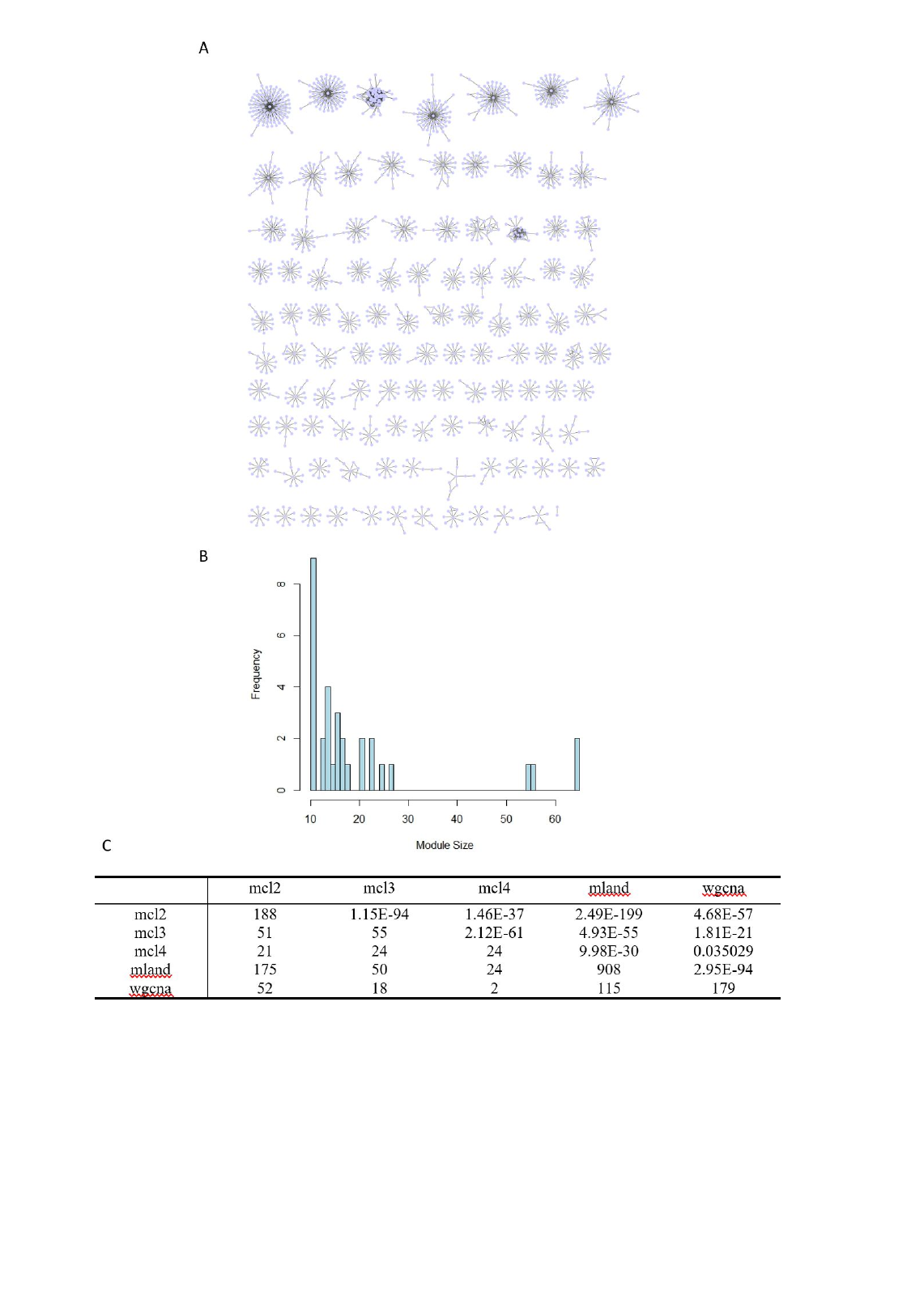

## Slide 5
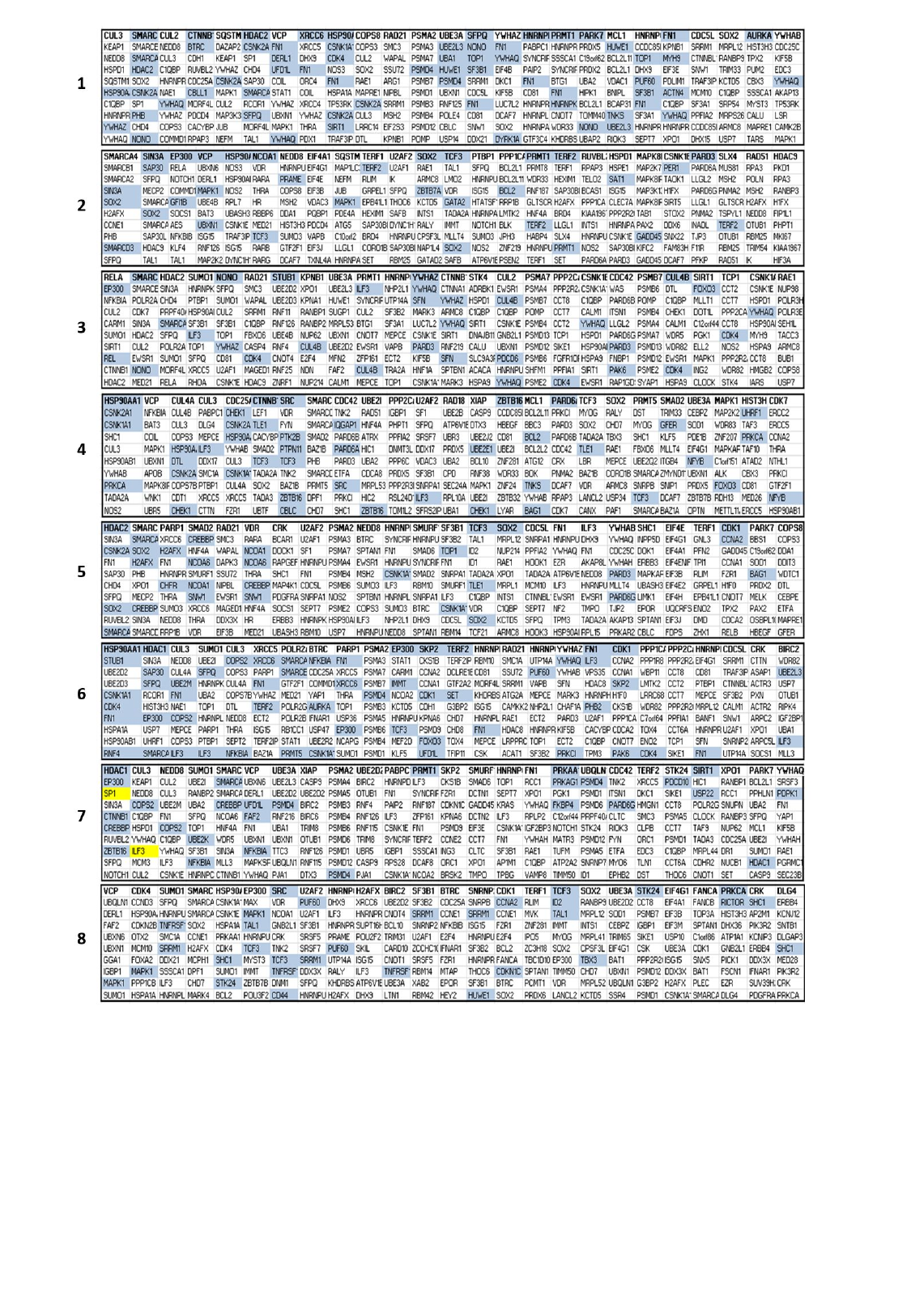

## Slide 6
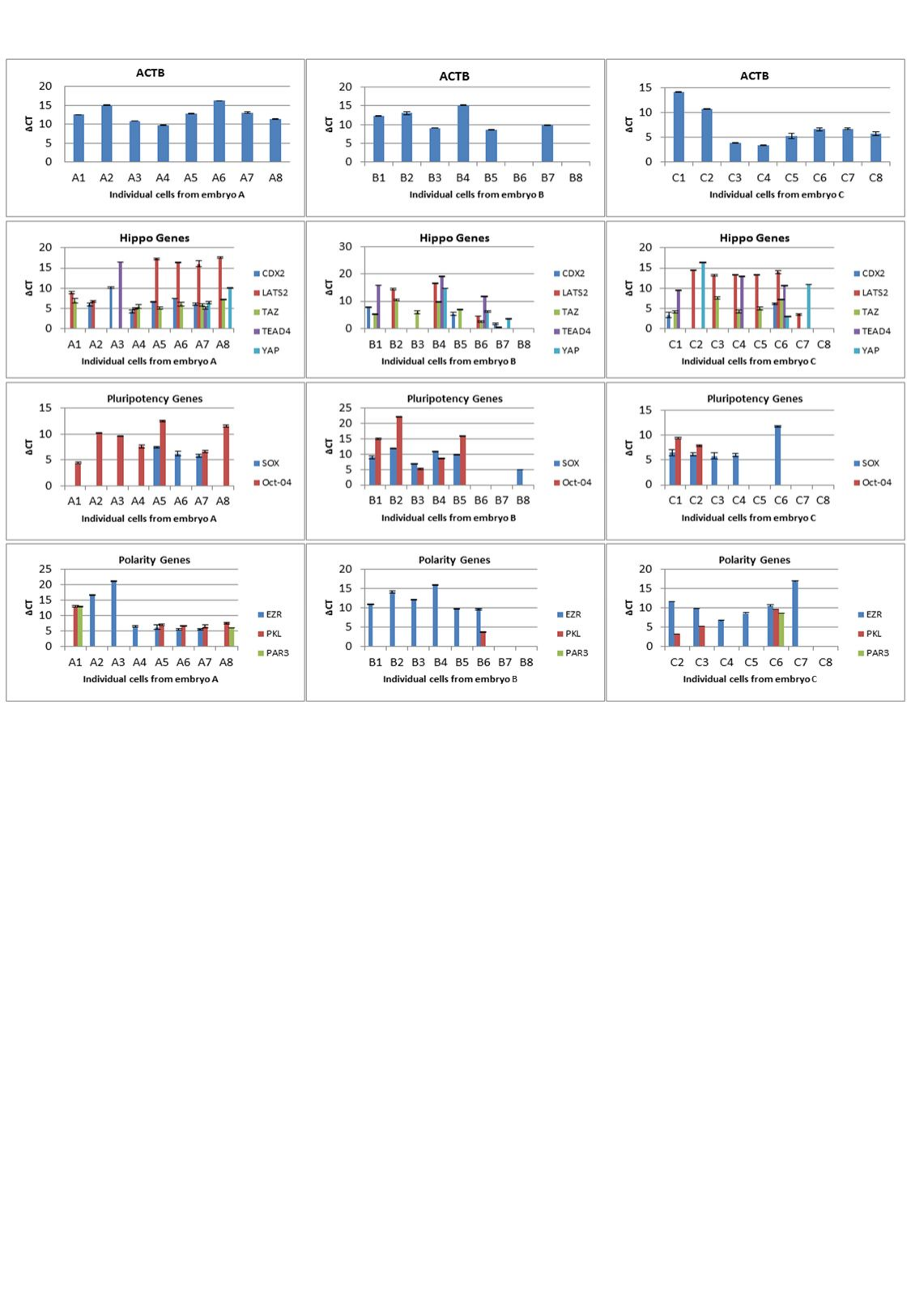

## Slide 7
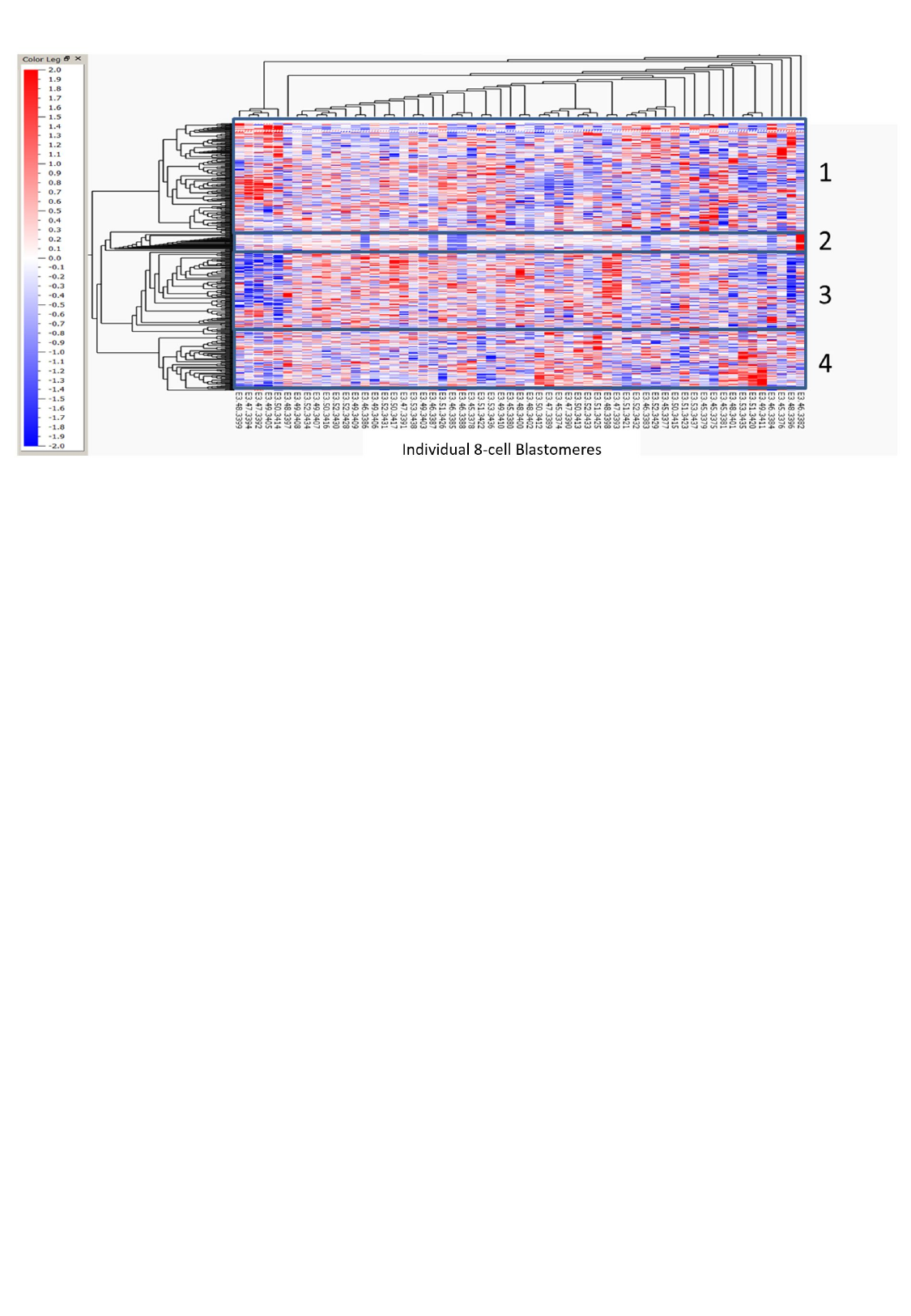

Supplement: Supplementary file 2 — Figure S1. Un-normalised Affymetrix microarrays A) Boxplots represent summaries of the signal intensity distributions of the arrays. Each box corresponds to one array. B) Boxplot outlier detection was performed by computing the Kolmogorov-Smirnov statistic Kabetween each array’s distribution and the distribution of the pooled data. None of the samples have medians higher than 1.05, which would represent a low quality array C) Density distributions of the log2intensities grouped by the matching type of the probes. The blue line shows a density estimate (smoothed histogram) from intensities of perfect match probes (PM), the grey line, one from the mismatch probes (MM). D) RNA digestion plot. The shown values are computed from the pre-processed data. Each array is represented by a single line. E) MA plots (M = log2 (I1)-log2 (I2), A = 1/2(log2 (I1) + log2 (I2)), where I1 is the intensity of the array studied, and I2 is the intensity of a “pseudo”-array that consists of the median across arrays. The mass of the distribution in an MA plot should be concentrated along the M = 0 axis, and there should be no trend in M as a function of A. Shown are first the 4 arrays with the highest values of Da, then the 4 arrays with the lowest values. F) An example of feature intensities representing the arrays’ spatial distributions (M). Figure S2. A and B) Embryonic genome activation (EGA) interaction networks, differential regulation within the 8-cell and blastocyst compared to the 4-cell. C and D) Metanodes of the 8-cell and blastocyst compared to the 4-cell, metanodes as defined by the Cytoscape plugin ‘Moduland’, metanodes represent genes most central within each module. Red genes represent up-regulation, green nodes represent down-regulation and pink nodes represent non-differentially regulated genes but baseline expressed direct interaction partners. E and F) Tables of network module members. Metanodes represent the most centrally connected gene within a module alongside the nex [file 12864_2019_5558_MOESM2_ESM.pptx]
